# The voltage‐gated calcium channel CaV1.2 promotes adult oligodendrocyte progenitor cell survival in the mouse corpus callosum but not motor cortex

**DOI:** 10.1002/glia.23723

**Published:** 2019-10-12

**Authors:** Kimberley A. Pitman, Raphael Ricci, Robert Gasperini, Shannon Beasley, Macarena Pavez, Jac Charlesworth, Lisa Foa, Kaylene M. Young

**Affiliations:** ^1^ Menzies Institute for Medical Research University of Tasmania Hobart Australia; ^2^ School of Medicine University of Tasmania Hobart Australia

**Keywords:** apoptosis, Cacna1C, calcium, CaV1.2, corpus callosum, motor cortex, NG2, oligodendrocyte, proliferation, survival, voltage‐gated calcium channel

## Abstract

Throughout life, oligodendrocyte progenitor cells (OPCs) proliferate and differentiate into myelinating oligodendrocytes. OPCs express cell surface receptors and channels that allow them to detect and respond to neuronal activity, including voltage‐gated calcium channel (VGCC)s. The major L‐type VGCC expressed by developmental OPCs, CaV1.2, regulates their differentiation. However, it is unclear whether CaV1.2 similarly influences OPC behavior in the healthy adult central nervous system (CNS). To examine the role of CaV1.2 in adulthood, we conditionally deleted this channel from OPCs by administering tamoxifen to P60 *Cacna1c* ^*fl*/*fl*^ (control) and *Pdgfrα‐CreER*:: *Cacna1c* ^*fl*/*fl*^ (CaV1.2‐deleted) mice. Whole cell patch clamp analysis revealed that CaV1.2 deletion reduced L‐type voltage‐gated calcium entry into adult OPCs by ~60%, confirming that it remains the major L‐type VGCC expressed by OPCs in adulthood. The conditional deletion of CaV1.2 from adult OPCs significantly increased their proliferation but did not affect the number of new oligodendrocytes produced or influence the length or number of internodes they elaborated. Unexpectedly, CaV1.2 deletion resulted in the dramatic loss of OPCs from the corpus callosum, such that 7 days after tamoxifen administration CaV1.2‐deleted mice had an OPC density ~42% that of control mice. OPC density recovered within 2 weeks of CaV1.2 deletion, as the lost OPCs were replaced by surviving CaV1.2‐deleted OPCs. As OPC density was not affected in the motor cortex or spinal cord, we conclude that calcium entry through CaV1.2 is a critical survival signal for a subpopulation of callosal OPCs but not for all OPCs in the mature CNS.

## Main points

- OPCs express CaV1.2 in development and adulthood.
- The conditional deletion of CaV1.2 from adult OPCs enhances their proliferation.
- Deleting CaV1.2 from adult OPCs has no effect on oligodendrogenesis or myelination.
- Loss of CaV1.2 kills OPCs in the corpus callosum but these cells are rapidly replaced by surviving parenchymal OPCs that are not reliant on CaV1.2 for survival.

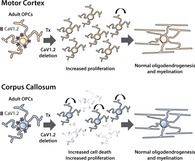

## 1 INTRODUCTION

Oligodendrocyte progenitor cells (OPCs) are immature, proliferative cells that differentiate into mature, myelinating oligodendrocytes in the central nervous system (CNS). OPCs generate large numbers of oligodendrocytes shortly after birth and continue to divide and generate new oligodendrocytes throughout life (reviewed by Richardson, Young, Tripathi, and McKenzie (2011)). They express receptors and ion channels that allow them to detect and respond to neuronal activity (reviewed by Larson, Zhang, and Bergles (2015)), and it is well established that neuronal activity can modulate OPC behavior, influencing migration, proliferation and differentiation (reviewed by Wang and Young (2014), Fields (2015), and Purger, Gibson, and Monje (2015)). One category of ion channel that is expressed by OPCs is the voltage‐gated calcium channels (VGCCs; Borges, Wolswijk, Ohlemeyer, & Kettenmann, 1995; Haberlandt et al., 2011). As VGCCs are important for the ability of other cell types to translate an activity‐induced change in membrane potential into a calcium‐dependent response, such as the release of neurotransmitters or altered gene expression (reviewed by Striessnig et al. (2006)), calcium entry through VGCCs on OPCs may similarly transduce activity‐dependent changes in OPC behavior (reviewed by Pitman and Young (2016)).

OPCs predominantly express two classes of VGCCs, the L‐type and T‐type channels (Fulton et al., 2010; Haberlandt et al., 2011). The L‐type channel CaV1.2, encoded by the gene *Cacna1c*, is the major channel responsible for depolarization‐induced calcium entry into developmental OPCs, as measured by calcium imaging in vitro (Cheli et al., 2016; Cheli, Santiago González, Spreuer, & Paez, 2015) and ex vivo (Cheli et al., 2016). The conditional deletion of CaV1.2 from OPCs in the early postnatal mouse CNS leads to hypomyelination of the corpus callosum and cortex (Cheli et al., 2016). While OPC proliferation was reduced in the corpus callosum, and brain slice culture experiments suggested that CaV1.2 was also required for OPC migration, the hypomyelination was largely attributed to the impaired ability of OPCs to generate myelinating oligodendrocytes (Cheli et al., 2016).

Consistent with this finding, the deletion of CaV1.2 from adult OPCs was reported to reduce their capacity to remyelinate the corpus callosum following cuprizone‐induced demyelination (Santiago González et al., 2017), suggesting that CaV1.2 can, at least following an injury, regulate adult OPC function. However, it is also possible that CaV1.2 may differentially regulate developmental versus adult OPCs or OPCs in the healthy versus injured CNS. In mice, OPCs are known to undergo age‐related changes, including the slowing of proliferation with increasing postnatal age (Clarke et al., 2012; Young et al., 2013) and the reduction of voltage‐gated sodium channel expression between development (P6‐16) and adulthood (>P80) (Spitzer et al., 2019). Furthermore, a transcriptomics analysis of adult OPCs has indicated that they are more similar to oligodendrocytes than they are to neonatal OPCs (Moyon et al., 2015). Herein, we show that adult OPCs express L‐type VGCCs at similar levels to their developmental counterparts, and while the deletion of CaV1.2 from adult OPCs increases their proliferation, it does not affect oligodendrogenesis or myelination. Instead, the deletion of CaV1.2 triggers the apoptotic cell death and loss of approximately half of all adult OPCs from the corpus callosum without affecting the survival of OPCs located in the motor cortex or spinal cord. These data indicate that CaV1.2 is an essential survival signal for a subset of adult OPCs in the corpus callosum.

## 2 MATERIALS AND METHODS

### 2.1 Animals and housing

All animal experiments were approved by the University of Tasmania Institutional Biosafety Committee and Animal Ethics Committee (A0013741 and A0016151) and were carried out in accordance with the Australian code of practice for the care and use of animals for scientific purposes. All mice were maintained on a C57bl/6 background and housed in optimice microisolator cages (Animal Care Systems, Centennial, CO) on a 12‐hr light/dark cycle at 20°C. Male and female mice were weaned after P30, to allow normal myelin development, and housed with gender‐matched littermates. Food and water were available ad libitum. Heterozygous *Pdgfrα–H2BGFP* (*Pdgfrα‐hGFP*; Hamilton, Klinghoffer, Corrin, & Soriano, 2003), heterozygous *Pdgfrα‐CreER* (Kang, Fukaya, Yang, Rothstein, & Bergles, 2010), heterozygous *Tau‐lox‐STOP‐lox‐mGFP‐IRES‐NLS‐LacZ‐pA* (*Tau‐mGFP*; Hippenmeyer et al., 2005) and homozygous *Cacna1c floxed* (*Cacna1c* ^*fl*/*fl*^; Seisenberger et al., 2000) transgenic mice were purchased from Jackson Laboratories. Heterozygous *Pdgfrα‐CreER* ^*T2*^ (Rivers et al., 2008) transgenic mice were a kind gift from Prof William D Richardson (University College London). In text, CaV1.2‐deleted mice refer to mice that carry the *Pdgfrα‐CreER* or *Pdgfrα‐CreER* ^*T2*^ transgene and two floxed copies of the *Cacna1c* gene (*Cacna1c* ^*fl/fl*^). Control mice (CTRL) refer to mice that either carry the *Pdgfrα‐CreER* or *Pdgfrα‐CreER* ^*T2*^ transgene, but have a normal *Cacna1c* gene, or lack the *Pdgfrα‐CreER* or *Pdgfrα‐CreER* ^*T2*^ transgene, but carry the floxed *Cacna1c* gene (*Cacna1c* ^*fl/fl*^). These mouse lines were inter‐crossed at the University of Tasmania to generate the experimental mice used for this study.

Please note that two distinct *Pdgfrα‐CreER* transgenic mouse lines were used in this study, each maintained as a heterozygous stock line: the *Pdgfrα‐CreER* transgenic mouse line generated by Kang et al. (2010) was used for the majority of experiments, while the *Pdgfrα‐CreER* ^*T2*^ transgenic mouse line generated by Rivers et al. (2008) was used to perform the *Tau‐mGFP* lineage tracing experiments only (Figure 3). When either line is crossed with the *Rosa26‐YFP* cre‐sensitive reporter mouse (Srinivas et al., 2001) and Tamoxifen administered to adult offspring, they specifically label and enable the lineage tracing of OPCs within the CNS (O'Rourke et al., 2016; Rivers et al., 2008). As *Cacna1c* is located on the same chromosome as *Rosa26*, we instead crossed each *Pdgfrα‐CreER* line with the *Tau‐mGFP* reporter mouse line (Hippenmeyer et al., 2005) and administered Tamoxifen to the adult offspring. The *Pdgfrα‐CreER* ^*T2*^ (Rivers et al., 2008) cross resulted in the mGFP‐labeling of a subset of adult OPCs and the new oligodendrocytes they produce (as per Young et al., 2013 and Figure 3). By contrast, the *Pdgfrα‐CreER* (Kang et al., 2010) cross resulted in extensive and seemingly nonspecific mGFP labeling throughout the CNS (Figure S1) and prevented our use of this mouse line for lineage tracing.

### 2.2 Genotyping of transgenic mice

Ear biopsies were digested in 100 mM Tris–HCl, 5 mM EDTA, 200 mM NaCl, and 0.2% SDS containing 0.48 mg/mL proteinase K (ThermoFisher Scientific). The cellular and histone proteins were precipitated by exposure to 6 M ammonium acetate (Sigma) and incubation on ice. After centrifugation, the DNA was precipitated from the supernatant by exposure to isopropyl alcohol (Sigma). The DNA pellet was washed in 70% ethanol (Sigma), resuspended in sterile MilliQ water and used as template DNA to genotype the mice by polymerase chain reaction (PCR). The PCR was performed as a 25 μL reaction containing: 50–100 ng DNA, 0.5 μL of each primer (100 nmol/mL; Integrated DNA Technologies) and 12.5 μL GoTaq green master mix (Promega) in MilliQ water. The following primer combinations were used: *Cre* 5′ CAG GTC TCA GGA GCT ATG TCC AAT TTA CTG ACC GTA and *Cre* 3′ GGT GTT ATA AGC AAT CCC CAG AA; *GFP* 5′ CCC TGA AGT TCA TCT GCA CCA C, and *GFP* 3′ TTC TCG TTG GGG TCT TTG CTC; or *Cacna1c* 5′ CTC CCA CTG TTT GAG CCT GT and *Cacna1c* 3′ TGT CTG CAG GTG GCA TAG. The PCR amplification program consisted of: 94°C for 4 min, followed by 35 cycles of 94°C for 30 s, 60°C for 45 s, and 72°C for 60 s, and a final 10 min at 72°C. The DNA was separated by gel electrophoresis (1% wt/vol agarose in TAE containing SYBR‐safe from ThermoFisher) and imaged using an Image Station 4000M PRO gel system running Carestream software.

### 2.3 Tamoxifen administration

Tamoxifen (Tx; Sigma) was reconstituted to 40 mg/mL in corn oil and placed in a sonicating water bath for ≥1 hr until dissolved. From P60, mice received 300 mg Tx per kilogram body weight daily for four consecutive days by oral gavage, as previously described (O'Rourke et al., 2016; Young et al., 2013). Mice were analyzed at various timepoints and referred to, for example, as P60 + 7, which indicates that the mice were analyzed at P67, 7 days after the first dose of Tx at P60.

### 2.4 Tissue preparation and immunolabeling

Mice were terminally anaesthetized with an intraperitoneal (i.p.) injection of sodium pentobarbital (30 mg/kg; Ilium) and then perfusion‐fixed with 4% (wt/vol) paraformaldehyde (PFA; Sigma) in phosphate buffered saline (PBS). The brain and spinal cord were removed, and the brain placed in a 1 mm brain matrix (Kent Scientific) and sliced coronally to generate 2 mm tissue blocks. All tissue was postfixed in 4% PFA at room temperature for 90 min before being cryoprotected in 20% (wt/vol) sucrose (Sigma) in PBS at 4°C overnight. Tissue was subsequently placed in cryomolds containing Shandon Cryomatrix (ThermoFisher), frozen using liquid nitrogen and stored at −80°C.

For immunolabeling, 30 μm coronal cryosections of the brain (Bregma 0.62–1.18 mm) or transverse cryosections of the spinal cord were collected and processed as floating sections. The primary antibodies used were: goat anti‐PDGFRα (1:200; GeneTex); rabbit anti‐NG2 (1:200, Millipore); mouse anti‐CC1 (1:100 Calbiochem); rat anti‐GFP (1:2000; Nacalai Tesque), rabbit anti‐cleaved caspase‐3 (1:200, Abcam); and rabbit anti‐Iba1 (1:1,000; Synaptic Systems). Secondary antibodies including donkey anti‐goat (1:1,000), donkey anti‐rabbit (1:1,000), donkey anti‐mouse (1:1,000), and donkey anti‐rat (1:500) were conjugated to Alexa Fluor −488, −568, or −647 (Invitrogen). Nuclei were labeled using Hoechst 33342 (1:1,000; Invitrogen). Mouse anti‐CC1 was diluted in blocking solution containing 0.1% (vol/vol) Triton X‐100 and 10% fetal calf serum (FCS) in Tris buffered saline. All other antibodies were diluted in PBS blocking solution (0.1% [vol/vol] Triton X‐100 and 10% FCS in PBS). Cryosections were incubated with antibodies overnight at 4°C on an orbital shaker. After immunostaining, floating sections were mounted onto glass slides and the fluorescence was preserved by the application of fluorescent mounting medium (Dako) with the coverslip.

### 2.5 7, 5‐Ethynyl‐2′‐deoxyuridine development and detection

From P60 + 7, 5‐ethynyl‐2′‐deoxyuridine (EdU; Invitrogen) was administered to mice via their drinking water (0.2 mg/mL, as per Young et al. (2013)) for 3, 6, 12, or 24 days. EdU labeling was visualized using the Alexa Fluor‐647 Click‐iT EdU kit (Invitrogen). Floating sections were incubated for 15 min in 0.5% Triton X‐100 (vol/vol) in PBS at room temperature. Cryosections were then transferred into the EdU developing cocktail, incubated in the dark for 45 min then washed in PBS before undergoing immunolabeling as described above. Alternatively, EdU was reconstituted to 5 mg/mL in PBS and filter sterilized, before being administered to P60 + 10 control and CaV1.‐2 deleted mice as 2x i.p. injections (each 25 mg/kg), 2 hr apart. Mice were killed by cervical dislocation 2 hr after the final injection (4 hr labeling period) and 200 μm thick coronal brain slices (generated as detailed below for calcium imaging) were immersion fixed in 4% PFA (wt/vol) in PBS for 90 min, prior to EdU developing.

### 2.6 Microscopy

Low magnification (20×) confocal image z‐stacks (3 μm spacing) were acquired spanning each transverse spinal cord cryosection using a spinning disk confocal microscope (DSD2, Andor). Images were stitched (NIS‐Elements, Nikon) to produce a single image for analysis. All other confocal images were collected using an UltraView confocal microscope with Volocity Software (Perkin Elmer) with standard excitation and emission filters for DAPI (Hoechst 33342), FITC (Alexa Fluor‐488), TRITC (Alexa Fluor‐568), and CY5 (Alexa Fluor‐647). A minimum of five cryosections were imaged per mouse. A series of images were collected (20× air objective) to span the corpus callosum or motor cortex (3 μm z‐spacing), and images covering each region were stitched together using the Volocity software. Images for internode analyses were collected using a 40× air objective and 0.5 μm z‐steps. Cell number, protein colabeling, tissue area measurements, and internode number and length measurements were made manually from images using the ImageJ software (NIH; (Schindelin et al., 2012)) or Adobe Photoshop, by researchers blinded to the experimental condition.

### 2.7 Electrophysiology

Following cervical dislocation, developing (P10‐P15) or adult (P60‐P84) *Pdgfrα‐hGFP* mice were decapitated and their brains dissected into an ice‐cold sucrose solution containing: 75 mM sucrose, 87 mM NaCl, 2.5 mM KCl, 1.25 mM NaH_2_PO_4_, 25 mM NaHCO_3_, 7 mM MgCl_2_, and 0.95 mM CaCl_2_. Coronal vibratome slices (300 μm) were prepared using a Leica VT1200s vibratome and incubated at 31.7°C in artificial cerebral spinal fluid (ACSF) containing: 119 mM NaCl, 1.6 mM KCl, 1 mM NaH_2_PO4, 26.2 mM NaHCO_3_, 1.4 mM MgCl_2_, 2.4 mM CaCl_2_, and 11 mM glucose (300 ± 5 mOsm/kg), saturated with 95% O_2_/5% CO_2_. After 45 min, slices were transferred to ~21°C ASCF.

Recordings of L‐type VGCCs were undertaken based on protocols in Fulton et al. (2010). Slices were transferred to a bath constantly perfused with ~21°C ACSF (2 mL/min). Recording electrodes were prepared from glass capillaries and had a resistance of 3–6 MΩ when filled with an internal solution containing 125 mM Cs‐methanesulfonate, 4 mM NaCl, 3 mM KCl, 1 mM MgCl_2_, 8 mM HEPES, 9 mM EGTA, 10 mM phosphocreatine, 5 mM MgATP, and 1 mM Na_2_GTP, set to a pH of 7.2 with CsOH and an osmolarity of 290 ± 5 mOsm/kg. Whole cell patch clamp recordings of GFP^+^ cells in the corpus callosum or motor cortex were collected using a HEKA Patch Clamp EPC800 amplifier and pCLAMP 10.5 software (Molecular devices). Due to the high membrane resistance of OPCs (>1 GΩ), recordings were made without series resistance compensation. Cells were held at −50 mV and a series of voltage steps up to +30 mV applied to determine the presence of a voltage‐gated sodium channel current, the identity of which was confirmed by the bath application of tetrodotoxin (TTX, 500 nM; Abcam) to a subset of cells (current at baseline: −232 ± 33 pA; current in the presence of TTX: −21 ± 6 pA; n = 4, *p* = .0008, paired *t* test).

The perfusate was subsequently switched to a calcium recording solution containing 20 mM BaCl_2_, 125 mM choline Cl, 5 mM tetraethyl ammonium, 10 mM glucose, and 10 mM HEPES, set to a pH of 7.4 with CsOH and an osmolarity of 300 ± 5 mOsm/kg. To record L‐type currents, cells were held at −50 mV and a series of 500 ms voltage steps, from −60 to +30 mV, was applied using a P/N subtraction protocol. Recordings were taken once a minute for ≥5 min. During the first 2 min, the voltage‐gated sodium current was completely blocked and the VGCC current could be recorded, indicating sufficient solution exchange. To block L‐type VGCC currents, nifedipine (30 μM; Sigma) or nimodipine (10 μM; Sigma) was added to the calcium recording solution. To block VGCC currents, cadmium (100 μM; Sigma) was added to the calcium recording solution. Access resistance was measured before and after each recording and an access resistance >20 MΩ resulted in exclusion of that recording. Measurements were made from each data file using Clampfit 10.5 (Molecular Devices). The steady‐state current was measured during the last 100 ms of each voltage step. The data file that produced the largest inwards current in each cell was selected for analysis, and statistical tests were performed on the evoked current that had the largest negative value (peak inward current), regardless of voltage step. In experiments where multiple genotypes were analyzed, all recordings were collected and analyzed by researchers unaware of the mouse genotype.

### 2.8 Calcium imaging

10 days after Tx administration (Tx + 10), *Pdgfrα‐hGFP* (control) and *Pdgfrα‐hGFP*:: *Cacna1c* ^*fl/fl*^ (CaV1.2‐deleted) mice were killed by cervical dislocation and each brain dissected into an ice‐cold sucrose solution containing: 75 mM sucrose, 87 mM NaCl, 2.5 mM KCl, 1.25 mM NaH_2_PO_4_, 25 mM NaHCO_3_, 7 mM MgCl_2_, and 0.95 mM CaCl_2_. Coronal vibratome slices (200 μm) were prepared using a Leica VT1200s vibratome and incubated at ~32°C in ACSF containing: 119 mM NaCl, 1.6 mM KCl, 1 mM NaH_2_PO_4_, 26.2 mM NaHCO_3_, 1.4 mM MgCl_2_, 2.4 mM CaCl_2_, and 11 mM glucose (300 ± 5 mOsm/kg), saturated with 95% O_2_/5% CO_2_. After 45 min, brain slices were transferred onto slice culture inserts (Millipore) suspended over 1 mL of ~21°C ASCF (saturated with 95% O_2_/5% CO_2_). A total of 5 μL of DMSO/10% pluronic acid (ThermoFisher)/5 mg/mL Fura‐2AM (ThermoFisher) was added to the tissue surface and the brain slice was incubated at 37°C/5% CO_2_ for 20 min. Each slice was washed with ACSF before being transferred to the microscope slice chamber, and continually perfused with ACSF. After 3 min of baseline imaging, slices were exposed to a depolarizing (high K^+^) ACSF for 2 min, which contained: 74 mM NaCl, 50 mM KCl, 1 mM NaH_2_PO_4_, 26.2 mM NaHCO_3_, 1.4 mM MgCl_2_, 2.4 mM CaCl_2_, and 11 mM glucose (300 ± 5 mOsm/kg), saturated with 95% O_2_/5% CO_2_. Images were captured every 5 s for 10 min on an inverted microscope (TiE, Nikon), equipped with a 40× Flour‐S oil‐immersion objective with DIC optics (Nikon) and NIS Elements 6D software (Nikon). Fura‐2AM was excited at 340 and 380 nm with an attenuated illumination source (50% transmission; Lambda DG‐4; Sutter Instruments, Novato, CA). Images were acquired at 510 nm with an EMCCD digital camera (Evolve 512; Photometrics) using regions of interest defined by NIS Elements software (Nikon). After subtracting background fluorescence, a ratio (*F*) of fluorescence at 340 nm to fluorescence at 380 nm was calculated in the soma. Cells were subsequently classified as GFP^+^ or GFP‐negative. The Fura‐2AM ratio was normalized to the baseline average (first 2 min) from each trace (*F*_0_). The maximum Δ*F*/*F*_0_ and area under the curve (Δ*F*/*F*_0_. min^−1^) calculations were made using Prism (GraphPad Software).

### 2.9 Statistical analyses

Statistical comparisons were made using GraphPad Prism. Each data set was first analyzed for normality using the KS normality test. As our data passed the normality test, they were further analyzed using a *t* test or one‐ or two‐way analysis of variance (ANOVA) followed by a Bonferroni's multiple comparisons post hoc test as indicated. For electrophysiological recordings, data are presented as mean ± *SEM* and *n* represents the number of cells from a minimum of three mice. Internode analyses are presented as mean ± *SEM* and *n* indicate the number of oligodendrocytes. All other histological data are presented as mean ± *SD* and *n* represents the number of mice. The data that support the findings of this study are available from the corresponding author upon reasonable request.

## 3 RESULTS

### 3.1 CaV1.2 is the major L‐type VGCC expressed by adult OPCs

**Figure 1:**
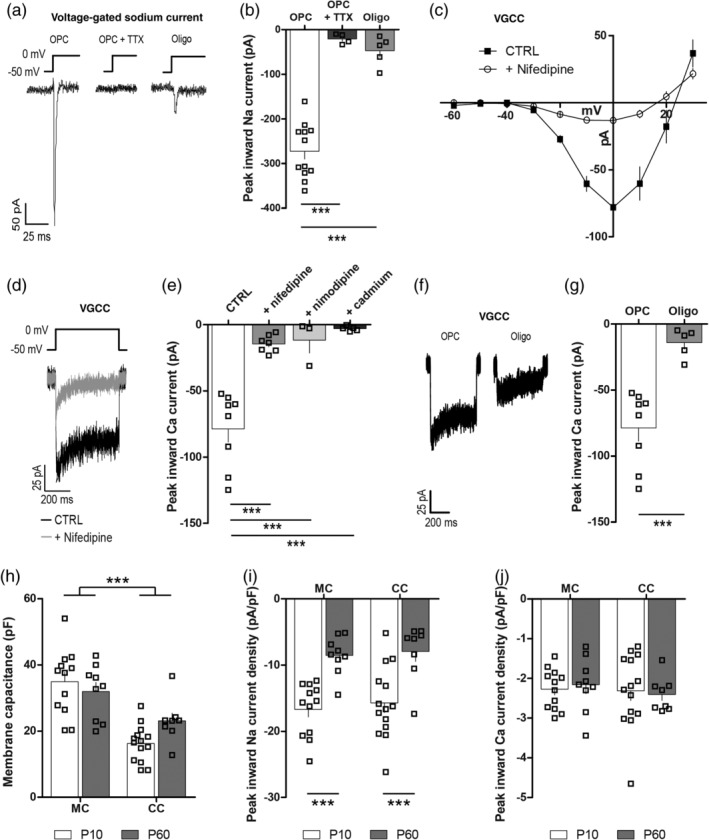
Adult oligodendrocyte progenitor cells (OPCs) express L‐type voltage‐gated calcium channel (VGCCs). Whole cell patch clamp recordings were made from GFP^+^ cells in acute brain slices from *Pdgfrα‐hGFP* transgenic mice. (a) Representative traces of voltage‐gated sodium channel currents evoked in a GFP^+^ OPC (in the absence and presence of tetrodotoxin [TTX]) or a newborn oligodendrocyte (oligo) by a depolarizing step from −50 to 0 mV. (b) Peak inward voltage‐gated sodium currents were measured in GFP^+^ cells in the absence or presence of TTX, and the magnitude of the evoked current was used to distinguish OPCs (*n* = 8) from oligos (*n* = 5). (c) Graphical representation of the current–voltage relationship for leak subtracted L‐type VGCC currents recorded from GFP^+^ OPCs in the P60 mouse motor cortex (MC) under control (CTRL) conditions (closed squares, *n* = 8) or in the presence of the L‐type VGCC antagonist nifedipine (30 μM; open circles, *n* = 7). (d) Representative traces depicting the current evoked by a depolarizing step from −50 to 0 mV in the absence (black) or presence (gray) of nifedipine. (e) The peak inward calcium current recorded from OPCs during a series of depolarizing steps under control conditions or in the presence of nifedipine, nimodipine, or cadmium. (f) Representative traces depicting the L‐type VGCC current evoked by a depolarizing step from −50 to 0 mV in a GFP^+^ OPC or oligo. (g) Peak inward L‐type VGCC calcium current measured in GFP^+^ OPCs or Oligos during a series of depolarizing steps. (h) The membrane capacitance (pF) of OPCs in the mouse corpus callosum (CC; P10, *n* = 14; P60, *n* = 8) or MC (MC; P10, *n* = 12; P60, *n* = 9). (i) Voltage‐gated sodium channel current density for OPCs from the MC or CC at P10 or P60. (j) L‐type VGCC current density for OPCs in the P10 and P60 MC or CC. Data are represented at mean ± *SEM*. ***p < .001. Data were analyzed using an unpaired *t* test (2 bar graphs), one‐way or two‐way analysis of variance (ANOVA) with Bonferroni's posttest

In order to determine whether CaV1.2 signaling regulates adult OPC function, we first confirmed that adult OPCs had functional L‐type VGCCs. We performed a whole cell patch clamp analysis of GFP‐labeled OPCs in the motor cortex of acute brain slices generated from P60 *Pdgfrα‐hGFP* mice (Figure 1). The majority of GFP^+^ cells were small cells with a membrane capacitance (Cm) <50 pF and a voltage‐gated sodium channel current >100 pA, which was sensitive to TTX (Figure 1a,b), confirming that they were OPCs. OPCs were held at −50 mV in a solution designed to isolate VGCC currents, and a series of voltage steps applied from −60 to 30 mV. OPCs responded with an inward current that appeared at approximately −30 mV, peaked at 0 mV, and was sensitive to the L‐type VGCC antagonists nifedipine (30 μM) or nimodipine (10 μM), or the nonspecific VGCC antagonist cadmium (100 μM) (Figure 1c–e). A minority of GFP^+^ cells had a Cm > 40 pF (mean 67 ± 12 pF) and an inward voltage‐gated sodium channel current <100 pA (Figure 1a,b) and were classified as newly differentiated oligodendrocytes (as per Clarke et al., 2012; Sahel et al., 2015). The magnitude of the inward VGCC current was smaller in newly differentiated oligodendrocytes than in OPCs (Figure 1f–g). These data indicate that, like developmental OPCs (Fulton et al., 2010; Hrvatin et al., 2018; Zhang et al., 2014), adult OPCs express L‐type VGCCs, that are downregulated as they differentiate into oligodendrocytes.

To determine if L‐type VGCC expression by OPCs differs with age or brain location, we measured the VGCC current in OPCs located in the motor cortex or corpus callosum of P10 or P60 mice. OPCs in the corpus callosum consistently had a smaller Cm than OPCs in the motor cortex (Figure 1h), suggesting that they are smaller. When the peak inward current was normalized to Cm for each cell (peak inward current/Cm = current density), we found that the magnitude of the OPC peak inward voltage‐gated sodium channel and L‐type VGCC currents were unaffected by brain region (Figure 1i,j). Furthermore, while voltage‐gated sodium channel current density was reduced in adult OPCs compared with developmental OPCs (Figure 1i), VGCC current density was equivalent for OPCs found in the P10 and P60 mouse brain (Figure 1j). These data suggest that brain OPCs maintain VGCC expression throughout development and into adulthood.

**Figure 2:**
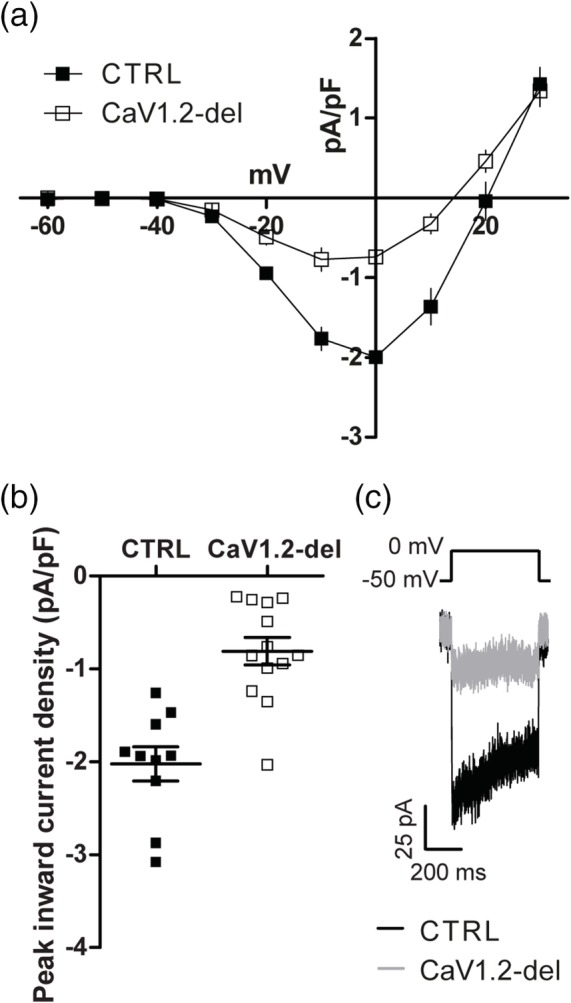
CaV1.2 is the major voltage‐gated calcium channel (VGCC) expressed by adult oligodendrocyte progenitor cells (OPCs). (a) Graphical representation of the current density‐voltage relationship for leak‐subtracted L‐type VGCC currents recorded from OPCs in the motor cortex of P60 + 14 control (filled squares, *n* = 10) or CaV1.2‐deleted (*Pdgfrα‐CreER* : :*Cacna1c* ^*fl*/*fl*^; open squares, *n* = 13) mice. (b) The peak inward current density of L‐type VGCC currents recorded from control (filled squares) or CaV1.2‐deleted (open squares) OPCs during a series of depolarizing voltage steps (*p* < .001, unpaired *t* test). (c) Representative traces showing the leak‐subtracted calcium current evoked in response to a depolarizing step from −50 to 0 mV. Values represent mean ± *SEM*

During development, CaV1.2 is the major channel contributing to depolarization‐induced calcium entry into OPCs (Cheli et al., 2016). To determine whether CaV1.2 is also the major L‐type channel in adult OPCs, we conditionally deleted CaV1.2 from OPCs in the adult mouse CNS. Tx was administered to P60 *Pdgfrα‐hGFP*:: *Cacna1c* ^*fl*/*fl*^ (Control) and *Pdgfrα‐CreER*:: *Pdgfrα‐hGFP*:: *Cacna1c* ^*fl*/*fl*^ (CaV1.2‐deleted) mice and L‐type VGCC currents recorded from GFP^+^ OPCs in the motor cortex of acute slices generated at P60 + 14. Tx administration to control mice did not alter OPC VGCC current density (no Tx control −2.1 ± 0.2 pA/pF vs. Tx control −2 ± 0.2 pA/pF; *p* = .7, unpaired *t* test). However, Tx administration to CaV1.2‐deleted mice reduced OPC VGCC current density to −0.8 ± 0.1 pA/pF, which equated to a ~60% reduction in the peak inward VGCC current density (Figure 2a–c). Furthermore, the conditional deletion of CaV1.2 from adult OPCs significantly reduced K^+^ depolarization‐induced calcium entry into GFP^+^ OPCs in acute brain slices, without impacting calcium entry into GFP‐negative cells (Figure S2). These data indicate that CaV1.2 is the major L‐type VGCC in adult OPCs.

When comparing the peak L‐type VGCC current density for control and CaV1.2‐deleted OPCs, we noted that the majority of control OPCs had a peak L‐type VGCC current density between −2.6 and −1.4 pA/pF (within 1 *SD* of the mean), while only 1 of the 13 OPCs examined in the CaV1.2‐deleted mice had a peak L‐type VGCC current density within this range (Figure 2a–c). Therefore, we conservatively estimate that the *Cacna1c* gene was recombined and deleted from ~92% of OPCs in these mice (12/13 × 100). This recombination efficiency is in line with that previously reported for adult *Pdgfrα‐CreER*:: *Rosa26‐YFP* mice that received an equivalent Tx treatment (O'Rourke et al., 2016). When CaV1.2 was instead deleted from OPCs using the less efficient *Pdgfrα‐CreER* ^*T2*^ transgenic mouse, the VGCC current density was reduced by ~29% (control OPCs −2.1 ± 0.1 pA/pF, *n* = 27; CaV1.2‐deleted OPCs −1.5 ± 0.2 pA/pF, *n* = 12; unpaired *t* test *p* = .001), suggesting that in these mice, the *Cacna1c* transgene was successfully deleted from ~45% of OPCs (29% current reduction/60% current reduction × 0.92). Again, this recombination efficiency is consistent with that previously reported for *Pdgfrα‐CreER* ^*T2*^:: *Rosa26‐YFP* mice that received an equivalent Tx treatment (Rivers et al., 2008).

### 3.2 CaV1.2 does not affect adult OPC differentiation or newborn oligodendrocyte myelination

**Figure 3:**
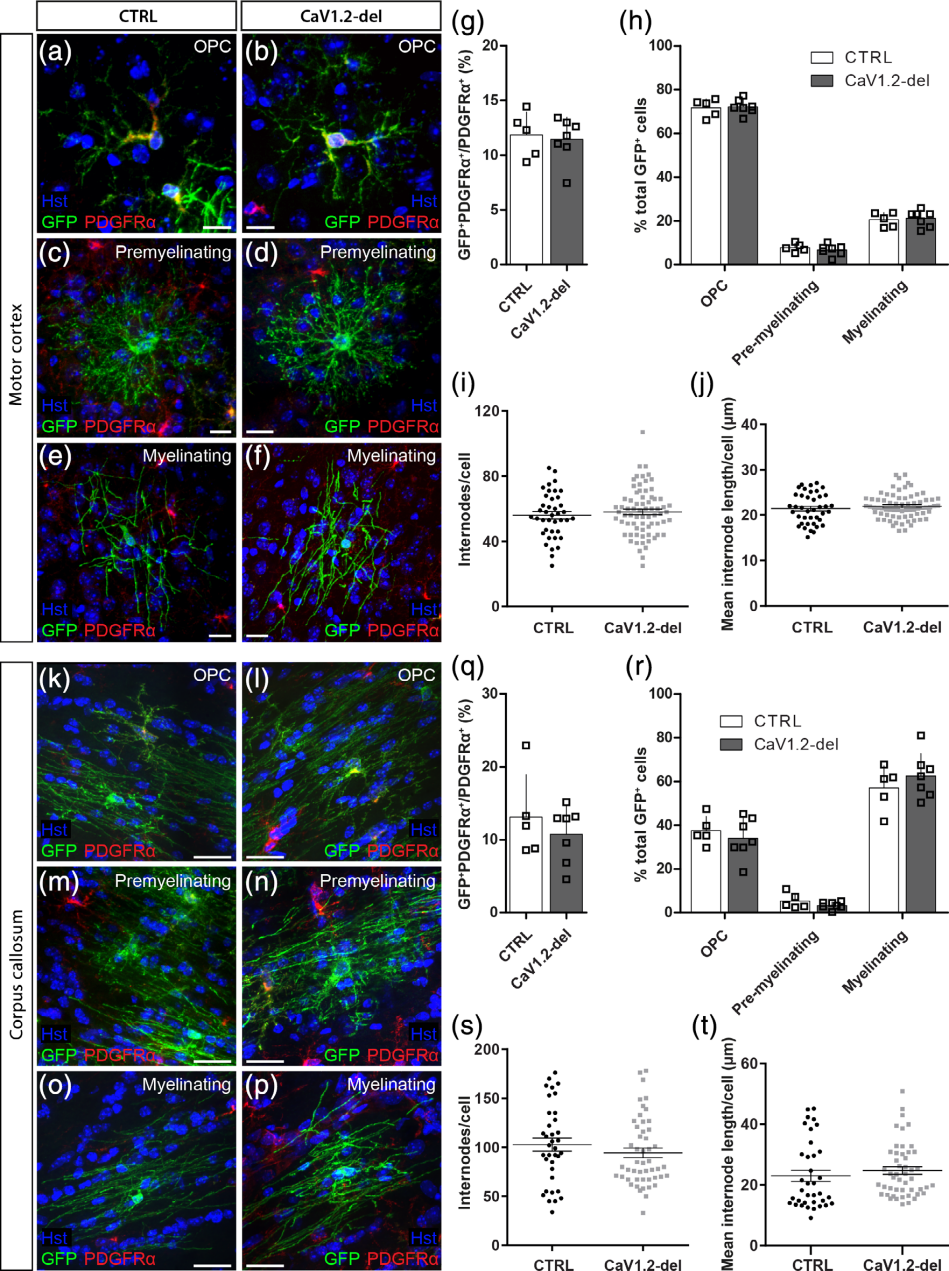
CaV1.2 is not required for oligodendrogenesis in the adult mouse brain. Brain cryosections from P60 + 30 control (CTRL) or CaV1.2‐deleted (*Pdgfrα‐CreER* ^*T2*^:: *Cacna1c* ^*fl/fl*^) mice were immunolabeled to detect membrane targeted (m) GFP (green) and PDGFRα (red). Representative images of mGFP^+^ PDGFRα^+^ oligodendrocyte progenitor cells (OPCs) (a,b), PDGFRα‐negative premyelinating oligodendrocytes (c,d), and PDGFRα‐negative myelinating oligodendrocytes (e,f) from the motor cortex (MC) of control and CaV1.2‐deleted mice. (g) The fraction of OPCs that recombine to express mGFP in the MC of control (*n* = 5) and CaV1.2‐deleted (*n* = 7) mice. (h) The proportion of mGFP^+^ cells in the MC that are OPCs, premyelinating oligodendrocytes or myelinating oligodendrocytes. (i) Average internode number per mGFP^+^ myelinating oligodendrocyte in the MC of control (*n* = 40 cells) and CaV1.2‐deleted (*n* = 71 cells) mice. (j) Average internode length per mGFP^+^ myelinating oligodendrocyte in the MC of control and CaV1.2‐deleted mice. Representative images of mGFP^+^ OPCs (k,l), premyelinating oligodendrocytes (m,n), and myelinating oligodendrocytes (o,p) in the corpus callosum (CC) of control or CaV1.2‐deleted mice. (q) The fraction of OPCs that recombine to express mGFP in the CC of control (*n* = 5) and CaV1.2‐deleted (*n* = 7) mice. (r) The proportion of mGFP^+^ cells in the CC that are OPCs, premyelinating oligodendrocytes or myelinating oligodendrocytes. (s) Average internode number per mGFP^+^ myelinating oligodendrocyte in the CC of control (*n* = 36 cells) and CaV1.2‐deleted (*n* = 49 cells) mice. (t) Average internode length per mGFP^+^ myelinating oligodendrocyte in the CC of control and CaV1.2‐deleted mice. Data are represented at mean ± *SD*, except for internode analyses, which are represented as mean ± *SEM*. Scale bars represent 10 μm [Color figure can be viewed at http://wileyonlinelibrary.com]

To determine whether CaV1.2 regulates OPC differentiation in the adult CNS, we performed lineage tracing of adult OPCs. Tx was administered to P60 *Pdgfrα‐CreER* ^*T2*^:: *Tau‐mGFP* (Control) and *Pdgfrα‐CreER* ^*T2*^:: *Tau‐mGFP*:: *Cacna1c* ^*fl/fl*^ (CaV1.2‐deleted) mice, to label adult OPCs with a membrane‐targeted form of GFP (mGFP) that is retained by the pre‐myelinating and myelinating oligodendrocytes they produce in the motor cortex (Figure 3a–j) and corpus callosum (Figure 3k–t). As the *Tau‐mGFP* transgene recombines inefficiently to label only a subset of OPCs in the adult mouse CNS, we performed immunohistochemistry to detect PDGFRα and mGFP in the P60 + 30 brain, and found that an equivalent fraction of PDGFRα^+^ OPCs were mGFP‐labeled in the motor cortex (Figure 3a,b,g) or corpus callosum (Figure 3k,l,q) of control and CaV1.2‐deleted mice.

To determine whether CaV1.2‐deletion altered the ability of adult OPCs to produce myelinating oligodendrocytes, we morphologically characterized the PDGFRα‐negative mGFP^+^ cells, classifying them as premyelinating (Figure 3c,d,m,n) or myelinating (Figure 3e,f,o,p) oligodendrocytes (as per Young et al. (2013)). In the motor cortex of P60 + 30 control and CaV1.2‐deleted mice, PDGFRα^+^ mGFP^+^ OPCs had differentiated to produce a small number of PDGFRα‐negative mGFP^+^ myelinating oligodendrocytes, that comprise ~20% of all mGFP^+^ cells in the region (Figure 3h). By contrast, PDGFRα^+^ mGFP^+^ OPCs in the corpus callosum of control and CaV1.2‐deleted mice had differentiated to produce many new mGFP^+^ myelinating oligodendrocytes that comprise ~60% of all callosal mGFP^+^ cells (Figure 3r). These data indicate that adult OPCs in the corpus callosum of control and CaV1.2‐deleted mice produce myelinating oligodendrocytes more rapidly than their cortical counterparts (*p* < .0001, unpaired *t* test). However, the number of mGFP^+^ myelinating oligodendrocytes was equivalent between control and CaV1.2‐deleted mice in each region (Figure 3h,r), indicating that CaV1.2 does not influence the number of OPCs that differentiate into myelinating oligodendrocytes in the adult mouse brain.

To determine whether CaV1.2 could influence the maturation of adult‐born oligodendrocytes, as reported in development (Cheli et al., 2016), we next examined the morphology of individual myelinating oligodendrocytes in the motor cortex (Figure 3e,f,i,j) and corpus callosum (Figure 3o,p,s,t) of P60 + 30 control and CaV1.2‐deleted mice. In the motor cortex, individual newborn, myelinating oligodendrocytes elaborated a smaller number of mGFP^+^ internodes than those added to the corpus callosum (compare control Figure 3i,s; *p* < .0001, unpaired *t* test). However, the deletion of CaV1.2 from adult OPCs did not affect the ability of newborn oligodendrocytes to produce the normal number of myelin internodes (Figure 3i,s), nor did it affect the length of the internodes produced (Figure 3j,t). These data suggest that adult‐born oligodendrocytes do not require CaV1.2 for myelination.

**Figure 4:**
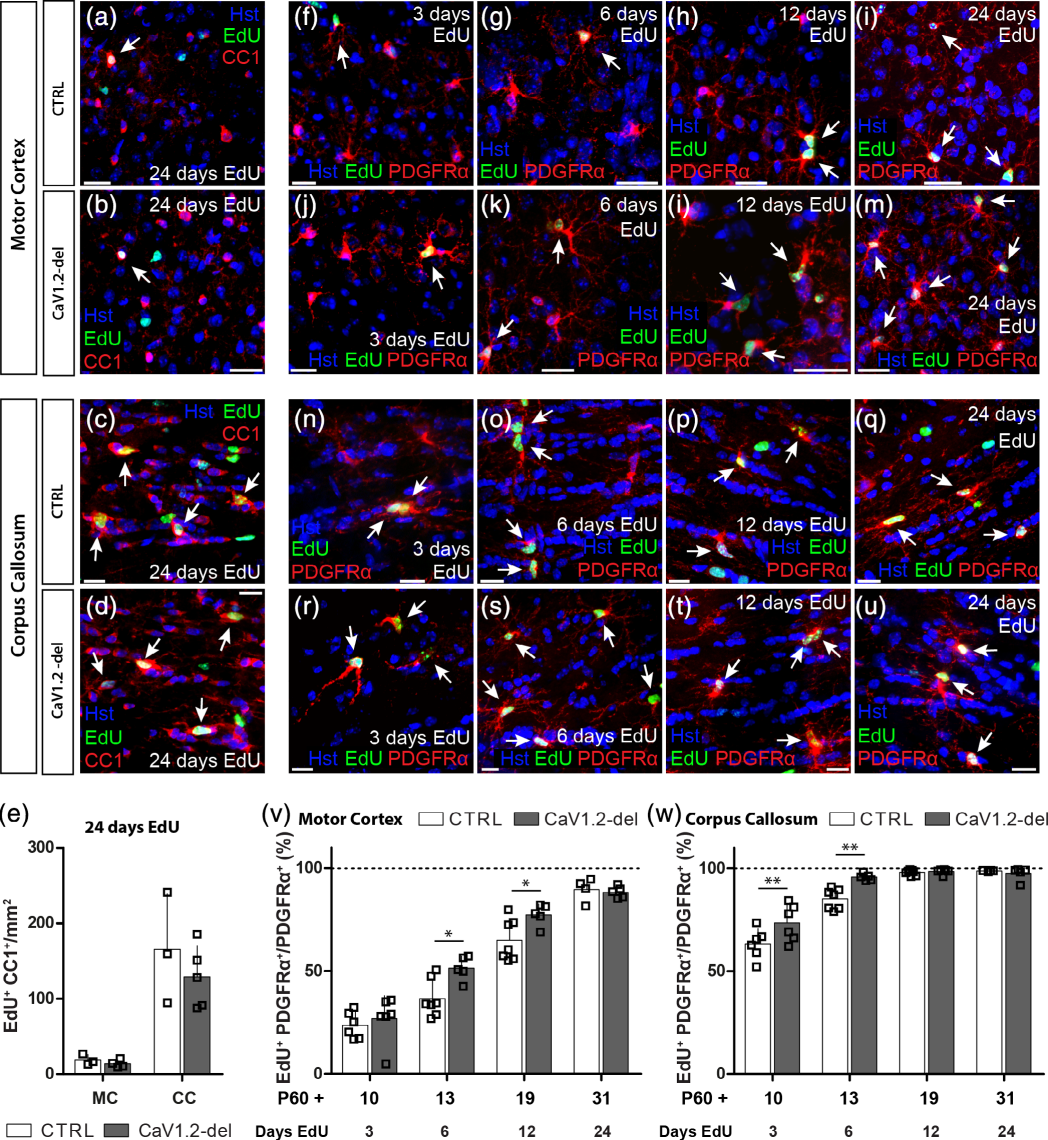
CaV1.2‐deletion increases adult oligodendrocyte progenitor cell (OPC) proliferation. Control (CTRL) and CaV1.2‐deleted (*Pdgfrα‐CreER*:: *Cacna1c* ^*fl/fl*^) mice received 7, 5‐ethynyl‐2′‐deoxyuridine (EdU) from P60 + 7 for up to 24 days before 30 μm brain cryosections were immunostained to detect EdU (green) and the oligodendrocyte marker CC1 (red) or the OPC marker PDGFRα (red). Representative images of newborn EdU‐labeled CC1+ oligodendrocytes in the motor cortex (MC) of control (a) and CaV1.2‐deleted (b) mice and the corpus callosum (CC) of control (c) and CaV1.2‐deleted (d) mice. (e) Quantification of the density (per mm^2^) of EdU^+^ CC1^+^ newborn oligodendrocytes in the MC and CC of control and CaV1.2‐deleted mice, after 24 consecutive days of EdU administration. Representative images of EdU‐labeled PDGFRα^+^ OPCs in the MC of control (f–i) and CaV1.2‐deleted (j–m) mice, and in the CC of control (n–q), and CaV1.2‐deleted (r–u) mice. (v) Quantification of the proportion of OPCs in the MC that incorporate EdU over time. (w) Quantification of the proportion of OPCs in the CC that incorporate EdU over time. Note that the EdU labeling of OPCs is saturated within 12 days of EdU delivery. Data are represented as the mean ± *SD* of *n* = 4–7 mice per genotype per timepoint. *p < .05 and **p < .01, two‐way analysis of variance (ANOVA) with Bonferroni's posttest. Scale bars represent 20 μm [Color figure can be viewed at http://wileyonlinelibrary.com]

As *Pdgfrα‐CreER* ^*T2*^:: *Cacna1c* ^*fl/fl*^ mice only delete CaV1.2 from approximately half of adult OPCs (see above), we confirmed this phenotype using *Pdgfrα‐CreER*:: *Cacna1c* ^*fl/fl*^ transgenic mice. We administered EdU to P60 + 7 control and *Pdgfrα‐CreER*:: *Cacna1c* ^*fl/fl*^ (CaV1.2‐deleted) mice for 24 consecutive days, and performed immunohistochemistry to detect EdU and the oligodendrocyte marker CC1 in the motor cortex (Figure 4a,b) and corpus callosum (Figure 4c,d). Consistent with our lineage tracing data (Figure 3), an equivalent density of EdU‐labeled CC1^+^ newborn oligodendrocytes accumulated in the motor cortex and corpus callosum of control and CaV1.2‐deleted (*Pdgfrα‐CreER*:: *Cacna1c* ^*fl/fl*^) mice during the labeling period (Figure 4e), confirming that CaV1.2 expression by OPCs does not impact oligodendrogenesis in adult mice.

### 3.3 CaV1.2 suppresses adult OPC proliferation

To examine the influence that CaV1.2 signaling exerts on adult OPC proliferation, we provided EdU to P60 + 7 control and *Pdgfrα‐CreER*:: *Cacna1c* ^*fl/fl*^ (CaV1.2‐deleted) mice, via the drinking water for up to 24 days, and performed immunohistochemistry to detect EdU and PDGFRα in the motor cortex (Figure 4f–m) and corpus callosum (Figure 4n–u). After 6 and 12 days of EdU delivery (P60 + 13 or P60 + 19), we detected a small but significant increase in the proportion of EdU‐labeled PDGFRα^+^ OPC (EdU^+^ PDGFRα^+^/total PDGFRα^+^; Figure 4v) in the motor cortex of CaV1.2‐deleted mice, relative to control mice (Figure 4v). More specifically, we found that the conditional deletion of CaV1.2 from adult OPCs significantly increased OPC proliferation in the superficial layers of the motor cortex (Figure S3). Furthermore, by providing EdU for a longer time period (24 days), we determined that CaV1.2 signaling in OPCs influenced their rate of proliferation, but did not influence the total proportion of OPCs capable of dividing (the proliferative fraction), as essentially all OPCs had become EdU‐labeled by P60 + 31 in control and CaV1.2‐deleted mice (Figure 4v).

CaV1.2 similarly suppresses OPC proliferation in the corpus callosum of adult mice, as the fraction of callosal OPCs that incorporated EdU over a 3 (P60 + 10) or 6 (P60 + 13) day labeling period was elevated in CaV1.2‐deleted mice, compared to control mice (Figure 4w). Consistent with this finding, the acute delivery of EdU to P60 + 10 mice (by i.p. injection), to label OPCs that proliferate within a 4‐hr window, revealed that approximately twice as many OPCs incorporated EdU in CaV1.2‐deleted mice than control mice (Figure S4). However, CaV1.2 did not influence the proportion of OPCs capable of dividing in the corpus callosum, as essentially all OPCs were EdU‐labeled following 12 or 24 days of EdU delivery to mice of either genotype (P60 + 19 and P60 + 31; Figure 4w). Therefore, we conclude that CaV1.2 reduces the rate of OPC proliferation in the healthy adult mouse motor cortex and corpus callosum but does not impact the fraction of OPCs that are capable of division.

### 3.4 CaV1.2 is essential for the survival of OPCs in the adult mouse corpus callosum, but not the motor cortex or spinal cord

**Figure 5:**
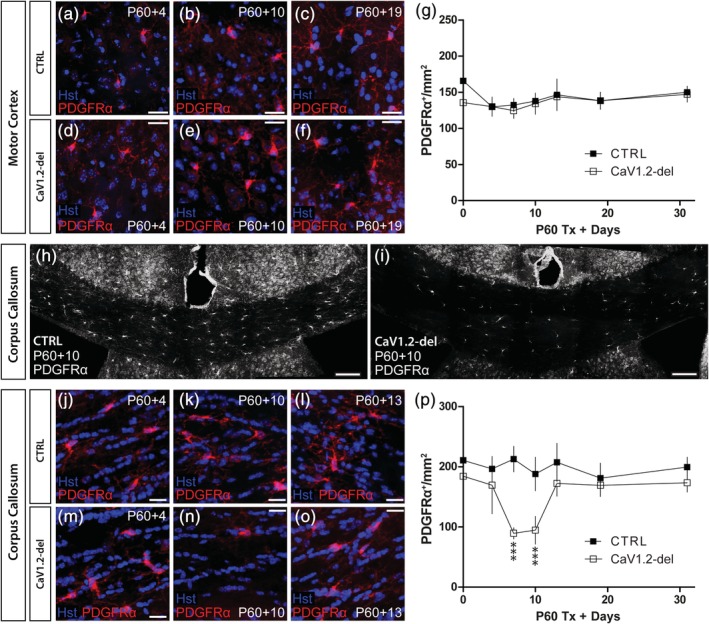
CaV1.2‐deletion leads to oligodendrocyte progenitor cells (OPCs) loss in the corpus callosum (CV) of adult mice. 30 μm brain cryosections were collected from control and CaV1.2‐deleted (*Pdgfrα‐CreER*:: *Cacna1c* ^*fl/fl*^) mice spanning P60 + 0 to P60 + 30 and stained to detect PDGFRα (red). Images show OPCs in the motor cortex (MC) of control (a–c) or CaV1.2‐deleted mice (d–f). (g) Quantification of the number of PDGFRα^+^ OPCs per mm^2^ (*x*‐*y*) in the MC of control (closed squares) and CaV1.2‐deleted (open squares) mice. (h) Low magnification image of the CC of a P60 + 10 control mouse stained to detect PDGFRα^+^ OPCs. (i) Low magnification image of the CC of a P60 + 10 CaV1.2‐deleted mouse stained to detect PDGFRα^+^ OPCs. Images show OPCs in the CC of control (j–l) or CaV1.2‐deleted (m–o) mice. (p) Quantification of the number of PDGFRα^+^ OPCs per mm^2^ (*x*‐*y*) in the CC of control (closed squares) and CaV1.2‐deleted (open squares) mice (genotype effect at P60 + 7 and P60 + 10; *p* < .001, two‐way analysis of variance (ANOVA) with Bonferroni's posttest). Data are represented as the mean ± *SD* of *n* = 4–7 mice per genotype per timepoint. Scale bars represent 20 μm (a–f, j–o) or 50 μm (h–i) [Color figure can be viewed at http://wileyonlinelibrary.com]

To investigate the possibility that CaV1.2 could regulate OPC number in the brain, we quantified the density of PDGFRα^+^ OPCs in the motor cortex (Figure 5a–g) and corpus callosum (Figure 5h–p) of control and CaV1.2‐deleted (*Pdgfrα‐CreER*:: *Cacna1c* ^*fl/fl*^) mice. We found that the density of PDGFRα^+^ OPCs did not vary significantly between P60 + 4 and P60 + 30 in the motor cortex of control or CaV1.2‐deleted mice and was equivalent to the OPC density of mice that did not receive tamoxifen (plotted as P60 + 0; Figure 5g). OPC density was also stable over time in the corpus callosum of control mice but OPC density dropped significantly between P60 + 4 and P60 + 7 in the corpus callosum of CaV1.2‐deleted mice, such that at P60 + 7, the density of PDGFRα^+^ OPCs was only ~42% that of control mice (Figure 5p). OPC density remained low in the corpus callosum of P60 + 10 CaV1.2‐deleted mice but returned to control levels by P60 + 13 (Figure 5p). NG2^+^ cell density was similarly reduced in the corpus callosum of P60 + 10 CaV1.2‐deleted mice, when compared to control mice (Figure S4e–g), confirming that CaV1.2 deletion resulted in OPC loss rather than the loss of PDGFRα expression by OPCs. As this reduction in OPC density was not recapitulated by the partial pharmacological blockade of CaV1.2, achieved by giving daily injections of nimodipine (as per Schampel et al. (2017)) for up to seven consecutive days (Figure S5), it is likely that OPC density can only be compromised by the complete and sustained loss of CaV1.2 signaling, as was achieved by our gene deletion approach.

**Figure 6:**
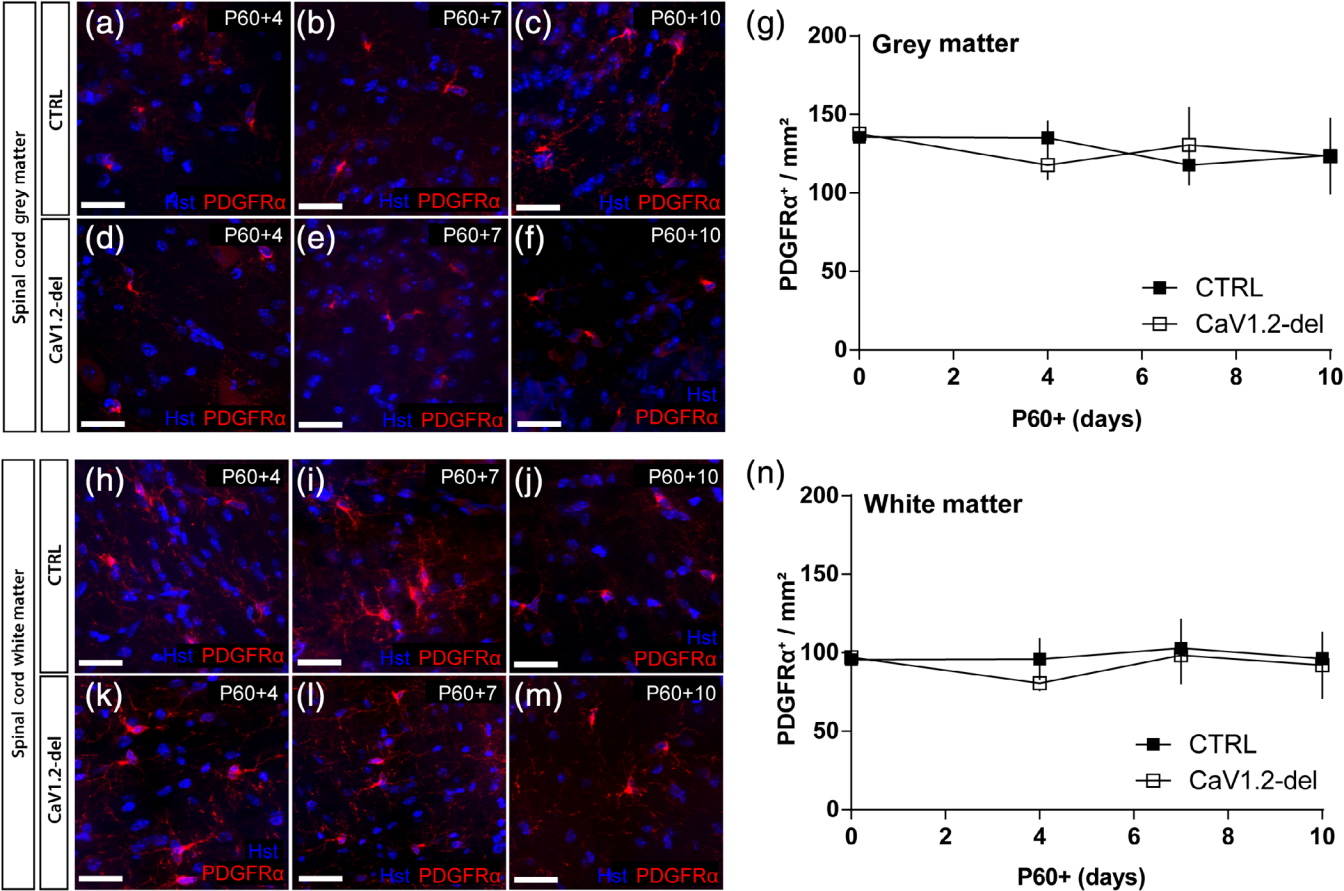
CaV1.2 does not influence oligodendrocyte progenitor cell (OPC) density in the spinal cord. 30 μm spinal cord (SC) cryosections from control and CaV1.2‐deleted (*Pdgfrα‐CreER*:: *Cacna1c* ^*fl/fl*^) mice aged P60 + 0 to P60 + 10 were immunostained to detect PDGFRα (red). Representative images of PDGFRα^+^ OPCs in the spinal cord gray matter of control (a–c) and CaV1.2‐deleted (d–f) mice. (g) Quantification of the number of PDGFRα^+^ OPCs per mm^2^ (*x*‐*y*) in the spinal cord gray matter of control (closed squares) and CaV1.2‐deleted (open squares) mice. Representative images of PDGFRα^+^ OPCs in the spinal cord white matter of control (h–j) and CaV1.2‐deleted (k–m) mice. (n) Quantification of the number of PDGFRα^+^ OPCs per mm^2^ (*x*‐*y*) in the spinal cord white matter of control (closed squares) and CaV1.2‐deleted (open squares) mice. Data are represented as the mean ± *SD* of *n* = 3–6 mice per genotype and timepoint. Scale bars represent 20 μm [Color figure can be viewed at http://wileyonlinelibrary.com]

To determine whether CaV1.2 affects OPC number in other white matter regions of the CNS, we performed immunohistochemistry to detect PDGFRα^+^ OPCs in the spinal cord gray (Figure 6a–g) and white matter (Figure 6h–n) of control and CaV1.2‐deleted (*Pdgfrα‐CreER*:: *Cacna1c* ^*fl/fl*^) mice. OPC density was higher in the spinal gray matter relative to the spinal cord white matter in mice of both genotypes (compare Figure 6g with Figure 6n; *p* < .001 regional affect, two‐way ANOVA), and OPC density was not affected by CaV1.2 expression in either spinal cord region between P60 + 0 and P60 + 10 (Figure 6g,n), the period corresponding with OPC loss from the corpus callosum. These data suggest that CaV1.2 supports the survival of a subset of OPCs in the corpus callosum, but that CaV1.2 is not critical for the survival of OPCs in all white matter regions of the CNS.

### 3.5 Callosal OPCs die by apoptosis and are replaced by parenchymal OPCs that lack CaV1.2

**Figure 7:**
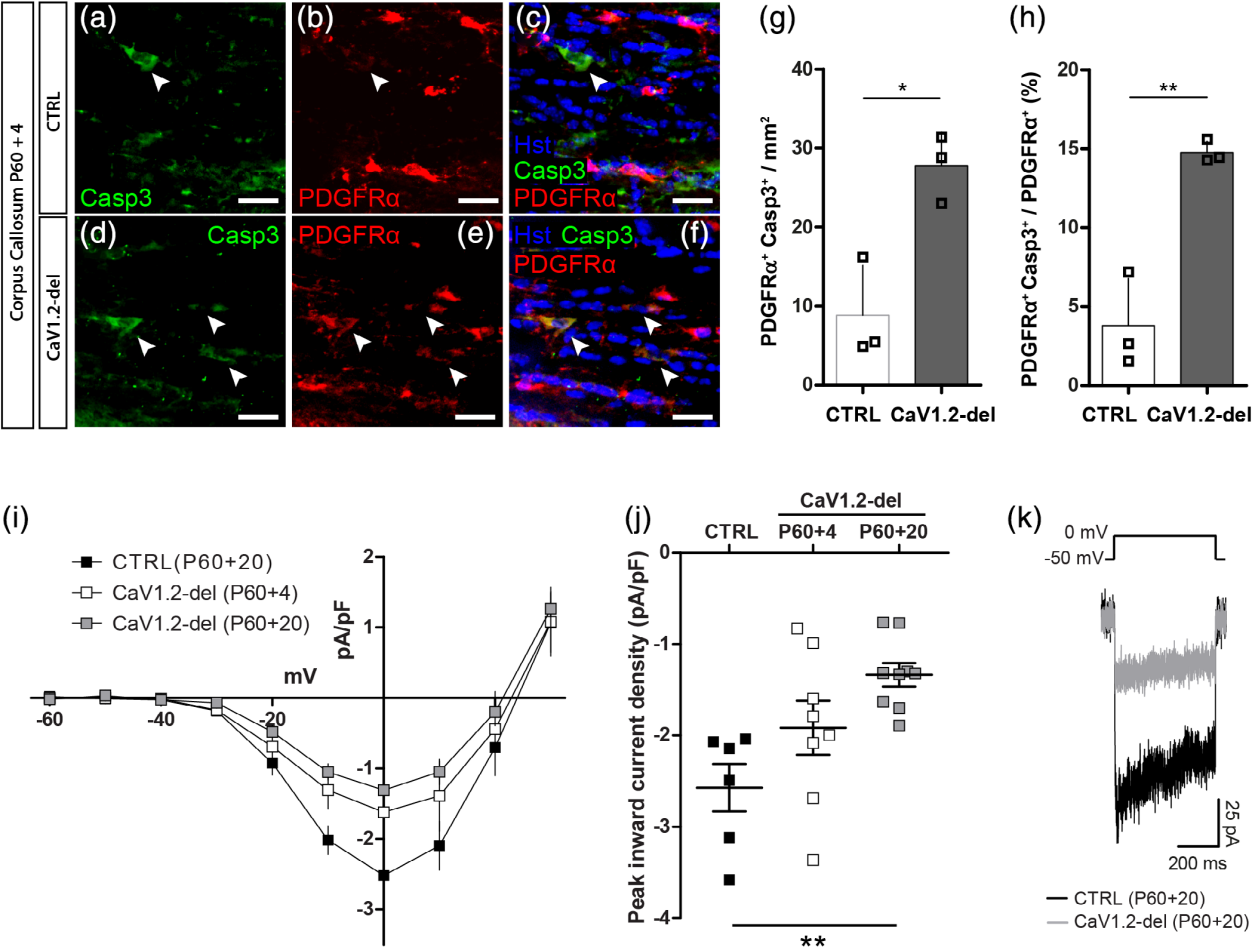
A subset of CaV1.2‐deleted callosal oligodendrocyte progenitor cells (OPCs) undergo caspase 3‐mediated apoptosis. (a–c) Representative image of the corpus callosum (CC) of a P60 + 4 control mouse stained to detect PDGFRα^+^ OPCs (red) and the apoptotic marker cleaved caspase 3 (casp3; green). (d–f) Representative image of the CC of a P60 + 4 CaV1.2‐deleted (*Pdgfrα‐CreER*:: *Cacna1c* ^*fl/fl*^) mouse stained to detect PDGFRα^+^ OPCs (red) and the apoptotic marker cleaved caspase 3 (casp3; green). Arrow heads indicate colabeled cells. (g) Quantification of the density of Caspase3^+^ PDGFRα^+^ OPCs per mm^2^ (*x*‐*y*; 30 μm cryosections) of the CC in control (*n* = 3) and CaV1.2‐deleted (*n* = 3) mice. (h) Quantification of the proportion of OPCs that express caspase 3 in control or CaV1.2 deleted mice. (i) Graphical representation of the current density‐voltage relationship for L‐type voltage‐gated calcium channel (VGCC) currents recorded from GFP^+^ OPCs in the CC of acute brain slices from control (filled squares; *n* = 6) mice at P60 + 20, or CaV1.2‐deleted mice at P60 + 4 (open squares; *n* = 7) and P60 + 20 (gray squares; *n* = 8). (j) The peak inward L‐type VGCC current density recorded from OPCs in slices generated from control (filled squares) mice at P60 + 20, or CaV1.2‐deleted mice at P60 + 4 (open squares) or P60 + 20 (gray squares). (k) Example traces show the leak‐subtracted voltage‐gated calcium current in response to a depolarizing step from −50 to 0 mV in OPCs from P60 + 20 control or CaV1.2‐deleted mice. Histological data are represented as mean ± *SD* and electrophysiological data are represented as mean ± *SEM*. *p < .05, **p < .01, *t* test, one‐way, or two‐way analysis of variance (ANOVA) with Bonferroni's posttest. Scale bars represent 20 μm [Color figure can be viewed at http://wileyonlinelibrary.com]

To determine whether callosal OPC density decreases in CaV1.2‐deleted mice because OPCs undergo apoptotic cell death, we stained coronal brain cryosections from P60 + 4 control and *Pdgfrα‐CreER*:: *Cacna1c* ^*fl/fl*^ (CaV1.2‐deleted) mice to determine whether PDGFRα^+^ OPCs expressed the apoptotic marker, cleaved caspase 3 (Figure 7a–f). While OPC density was equivalent in the corpus callosum of P60 + 4 control and CaV1.2‐deleted mice (Figure 5p), the density of PDGFRα^+^ Caspase3^+^ OPCs had already risen in the corpus callosum of CaV1.2‐deleted mice to be >threefold higher than the basal level of apoptosis observed in controls (Figure 7g). The fraction of OPCs that expressed caspase 3 increased from ~4% in control mice to ~15% in CaV1.2‐deleted mice (Figure 7h). These data suggest that the death of CaV1.2‐deleted callosal OPCs occurs rapidly following CaV1.2 loss. When making whole cell patch clamp recordings from healthy OPCs in the corpus callosum, we found no statistically significant difference between the peak inward L‐type VGCC current density of control and CaV1.2‐deleted OPCs at P60 + 4 (Figure 7i,j), suggesting that many OPCs still have intact CaV1.2 at this early timepoint. However, approximately half of the CaV1.2‐deleted OPCs had a peak inward L‐type VGCC current density below that observed in any control OPC (Figure 7i–k), suggesting that a subset of OPCs have lost or are losing CaV1.2 at the time of increased apoptosis.

For OPC density to return to normal in the corpus callosum of CaV1.2‐deleted mice by P60 + 13, new OPCs must be generated from the surviving parenchymal OPCs and/or from nearby neural stem cells. As neural stem cells do not express *Pdgfrα*, CaV1.2 would not be deleted from these cells at the time of Tx administration, and complete replacement of the lost callosal OPCs by neural stem cells would result in approximately half of callosal OPCs having intact CaV1.2 expression in P60 + 20 mice. Conversely, if the lost callosal OPCs were replaced by surviving CaV1.2‐deleted parenchymal OPCs, all OPCs in the repopulated corpus callosum would lack CaV1.2 at P60 + 20. To examine the origin of the replacement cells, we performed a whole cell patch clamp analysis of OPCs at P60 + 20, and found that OPCs in the corpus callosum of CaV1.2‐deleted mice had a peak inward L‐type VGCC current ~52% smaller than that of control OPCs at the same timepoint (Figure 7i–k). Furthermore, the L‐type VGCC current density measured for each OPC patched in the corpus callosum of P60 + 20 CaV1.2‐deleted mice was smaller than any of the L‐type VGCC current densities measured from OPCs in control mice (within the range of −3.2 pA/pF to −1.9 pA/pF), suggesting that all OPCs sampled lacked CaV1.2. While these data do not exclude the possibility that neural stem cells from the subventricular zone contribute in some small way to the repopulation, the absence of a large subset of callosal OPCs with intact L‐type VGCC currents suggests that OPCs in the corpus callosum of P60 + 20 CaV1.2‐deleted mice are primarily the surviving CaV1.2‐deficient parenchymal OPCs and their progeny.

## 4 DISCUSSION

### 4.1 CaV1.2 is not required for oligodendrogenesis or myelination in the healthy adult CNS

OPCs express various receptors and ion channels that enable them to respond to changes in neuronal activity by increasing intracellular calcium (reviewed by Pitman and Young (2016)), however, there is limited research exploring how this could change as mice age. Our data indicate that adult OPCs express L‐type VGCCs, particularly CaV1.2 (Figures 1, 2, and S2) and, consistent with previous findings in developmental OPCs (Fulton et al., 2010; Hrvatin et al., 2018; Zhang et al., 2014), these channels are downregulated as they differentiate into oligodendrocytes (Figure 1). We report that the conditional deletion of *Cacna1c* (CaV1.2) from adult OPCs (Figure 2) increases OPC proliferation (Figure 4; Figures S3 and S4) but does not affect the number of newborn oligodendrocytes added to the mature brain (Figures 3 and 4), or the ability of newborn oligodendrocytes to myelinate (Figure 3). These data are not consistent with the phenotype produced by the knockdown or conditional deletion of *Cacna1c* from developmental OPCs, where it has been shown to inhibit OPC proliferation and differentiation (Cheli et al., 2015, 2016) and result in hypomyelination of the brain (Cheli et al., 2016). It is perhaps surprising that the deletion of CaV1.2 from developmental OPCs impairs oligodendrocyte maturation, when CaV1.2 is downregulated with differentiation, but may suggest that calcium entry through CaV1.2 into developmental but not adult OPCs can have a long‐lasting effect on intracellular signaling or lead to their secretion of prodifferentiation signals. Alternatively, CaV1.2 could become restricted to the processes of new oligodendrocytes in development, but not in adulthood, such that the associated voltage‐gated calcium signal cannot be readily detected at the soma but could still influence developmental myelination. For example, action potentials are known to produce local calcium signals in internodes to influence the rate of internode extension and retraction in the developing CNS (Baraban, Koudelka, & Lyons, 2018; Krasnow, Ford, Valdivia, Wilson, & Attwell, 2018); however, it is not clear whether this type of myelin regulation requires CaV1.2.

As the L‐type VGCC currents measured in OPCs were unchanged between development and adulthood (Figure 1), a change in the expression of CaV1.2 is unlikely to account for the switch in CaV1.2 function over this period. However, VGCCs are activated by changes in membrane voltage, and their function can be influenced by the altered expression or activation of a number of other ion channels, including voltage‐gated sodium and potassium channels (Sun, Matthews, Nicolas, Schoch, & Dietrich, 2016), as the expression of these channels decreases from development to adulthood (Clarke et al., 2012; Spitzer et al., 2019; present data), or ionotropic glutamate or GABA receptors (Haberlandt et al., 2011; reviewed by Pitman and Young (2016)). The altered expression of these signaling proteins in OPCs could cause CaV1.2 to be activated less frequently in the adult mouse brain and limit the ability of CaV1.2 to be a dominant signal regulating OPC differentiation. It is also possible that CaV1.2 activity is affected by changes in the way that OPCs communicate with neurons and other CNS cell types in the developing versus adult CNS.

OPCs can communicate with neurons via GABAergic and glutamatergic synapses (Bergles, Roberts, Somogyi, & Jahr, 2000; Lin & Bergles, 2004) that are lost with increasing postnatal age in some brain regions, including the rat caudal cerebellar peduncle (Gautier et al., 2015) and mouse somatosensory cortex (Velez‐Fort, Maldonado, Butt, Audinat, & Angulo, 2010). Neuronal activity‐induced changes in OPC behavior could also result from changes in other modes of communication, such as the non‐synaptic release of molecules from axons (Maldonado, Velez‐Fort, Levavasseur, & Angulo, 2013), the secondary release of signaling molecules from astrocytes (Hamilton, Vayro, Wigley, & Butt, 2010), or extrasynaptic signaling resulting from neurotransmitter spill‐over from neuron–neuron synapses (Velez‐Fort et al., 2010). While it is unclear how callosal OPC inputs and signaling can change with aging, it is interesting to note that demyelination restores neuron‐OPC synaptic communication in the rat caudal cerebellar peduncle (Gautier et al., 2015) and causes adult OPCs in the corpus callosum to revert to a transcriptional state similar to that of developmental OPCs (Moyon et al., 2015). Such changes could account for CaV1.2 having no effect on adult myelination in the healthy adult mouse brain but acting to expedite or enhance remyelination of the corpus callosum following cuprizone‐induced demyelination (Santiago González et al., 2017).

### 4.2 CaV1.2 is a survival signal for a subpopulation of callosal OPCs

A key finding of this study is that CaV1.2 is critical for the survival of approximately half of all OPCs in the adult mouse corpus callosum. At P60 + 4, when the density of OPCs in the corpus callosum of CaV1.2‐deleted mice was normal (Figure 5), a subset of CaV1.2‐deleted OPCs had already entered the apoptotic cell death pathway (Figure 7), and by P60 + 7, the density of OPCs in the corpus callosum of CaV1.2‐deleted mice was reduced by ~58% (Figure 5). By contrast, OPC density in the motor cortex remained normal. These data indicate that CaV1.2 differentially regulates the survival of adult OPCs, which is consistent with previous reports that OPCs respond heterogeneously to some survival and cell death signals. To give examples, G protein‐coupled receptor 17 (GPR17)‐positive OPCs are more sensitive to ATP‐induced cell death than GPR17‐negative OPCs in vitro (Ceruti et al., 2011), and developmental but not adult OPCs rely on poly (RDP‐ribose) polymerase (PARP) activity for their survival (Baldassarro, Marchesini, Giardino, & Calzà, 2017). Additionally, the chromatin remodeler, Ch7, selectively promotes the survival of noncycling OPCs in the developing mouse corpus callosum and cortex, by downregulating *p53* (Marie et al., 2018).

CaV1.2 could enhance OPC survival by modulating known OPC survival signals, such as the expression or activity of the growth factor receptors activated by PDGF‐AA (Barres et al., 1992a, 1992b), neurotrophin 3 (Kumar, Kahn, Dinh, & de Vellis, 1998) and ciliary neurotropic factor (Dell'Albani et al., 1998; Talbott et al., 2007). By increasing p65 NFκB expression, factors secreted by microglia can increase the efficacy of PDGF‐AA signaling on cultured OPCs (Nicholas, Wing, & Compston, 2001), and tenascin‐C also promotes the survival of OPCs growing on poly‐lysine, by allowing them to respond to PDGF‐AA (Garwood et al., 2004). In vivo, OPCs rely on the HMG domain transcription factors, Sox9 and Sox10, to maintain PDGFRα expression and prevent their apoptotic cell death (Finzsch, Stolt, Lommes, & Wegner, 2008). However, CaV1.2 also promotes the survival of neurons in the developing mouse (P0–P18) cortex (Heck et al., 2008), superior olivary complex (Ebbers et al., 2015) and hippocampus (Lee et al., 2016), and calcium entry through CaV1.2 may instead promote OPC survival by increasing the activity of the cyclic‐AMP response element binding protein (CREB), a transcription factor expressed by OPCs (Zhang et al., 2014), and a downstream mediator of the CaV1.2‐dependent survival of cortical neurons (Heck et al., 2008). Further research is required to uncover the mechanism by which CaV1.2 activity enhances the survival of a discrete population of adult OPCs in vivo.

#### 4.2.1 Do functional subpopulations of OPCs exist in the brain?

It is intriguing that adult OPCs in the motor cortex and corpus callosum express CaV1.2 at similar levels, and yet only a subset of OPCs in the corpus callosum are reliant on CaV1.2 for their survival. Previous studies have shown that OPCs in different CNS regions proliferate at different rates, with OPCs in the white matter proliferating more rapidly than OPCs in gray matter (Young et al., 2013; present data), and that OPCs can differentially respond to growth factors, with cortical and cerebellar white but not gray matter OPCs proliferating in response to PDGF‐AA, despite their equivalent expression of PDGFRα (Hill, Patel, Medved, Reiss, & Nishiyama, 2013). OPCs in the corpus callosum and motor cortex have been previously subdivided based on their mixed origins in the lateral ganglionic eminence or cortical neuroepithelium (Kessaris et al., 2006), and brain OPCs can also be subdivided based on their expression of GRP17 (Boda et al., 2011), S100β (Hachem et al., 2005; Vives, Alonso, Solal, Joubert, & Legraverend, 2003), or the transcription factor Ascl1 (Mash1; Battiste et al., 2007; Parras et al., 2007; Nakatani et al., 2013). However, a clear overlap between any of these known subsets and the OPCs that require CaV1.2 for survival seems unlikely, as OPCs in the cortex have a similarly mixed origin (Kessaris et al., 2006) and contain OPCs that express GPR17 (Boda et al., 2011), Ascl1 (Nakatani et al., 2013) and S100β (Young et al., 2010) that do not die following CaV1.2 deletion.

Perhaps callosal OPCs are selectively dependent on CaV1.2 for their survival because of the level of CaV1.2 signaling they experience within their local environment. We have shown that all OPCs in the healthy mouse brain express L‐type VGCCs (see control recordings in Figures 2 and 7), and that CaV1.2, as the major L‐type VGCC, enables calcium entry into OPCs following K^+^‐induced depolarization (Figure S2). Furthermore, we show that CaV1.2 signaling impacts OPCs proliferation (Figure 4) in the healthy adult mouse brain, suggesting that CaV1.2 is activated under physiological conditions. However, these experiments do not tell us how often OPCs within different regions of the CNS depolarize and activate L‐type VGCCs. High voltage activated VGCCs open when a cell is depolarized to > −40 mV, making it possible that a subset of callosal OPCs become reliant on CaV1.2 for their survival because they are more frequently depolarized and their L‐type VGCCs more frequently activated. In the CA1 stratum radiatum, stimulation mimicking physiological glutamatergic synaptic signaling was able to induce both dendritic and somatic VGCC‐dependent calcium transients in OPCs in brain slices from P7‐15 mice, providing evidence that synaptic signaling can depolarize OPCs and activate VGCCs (Sun et al., 2016). However, these calcium transients were largely mediated via low‐voltage activated channels (Sun et al., 2016), suggesting that, at least in the CA1 stratum radiatum of developing mice, high voltage activated L‐type VGCCs may not be activated in this way. At present, we do not know which physiological conditions or patterns of signaling allows OPCs to become sufficiently depolarized to activate L‐type VGCCs, although OPCs express a complement of ion channels that can enable membrane depolarization, such as voltage‐gated sodium channels (Clarke et al., 2012), ionotropic glutamate or GABA receptors (Haberlandt et al., 2011), or purinergic ion channels (Hamilton et al., 2010) that are likely sources of physiological L‐type VGCC activation.

Adult OPCs that do not rely on CaV1.2 for survival may instead rely on an alternative source of calcium. In addition to voltage‐gated calcium entry, OPCs can rely on store‐operated calcium channels (Paez et al., 2009); however, an increase in store‐operated calcium entry between development and adulthood would be predicted to increase the stromal interaction molecule 1‐mediated suppression of calcium entry via CaV1.2 and reduce the cell surface expression of CaV1.2 (Park, Shcheglovitov, & Dolmetsch, 2010; Wang et al., 2010), both of which would have reduced the OPC L‐type VGCC current. OPCs that do not rely on CaV1.2 for their survival may still rely on calcium entry through calcium‐permeable ionotropic receptors, including AMPA receptors (Haberlandt et al., 2011), NMDA receptors (Hamilton et al., 2010), and P2X purinergic receptors (Hamilton et al., 2010). Alternatively, neurotransmitters including glutamate (Haberlandt et al., 2011), ATP (Hamilton et al., 2010), and acetylcholine (Welliver et al., 2018) can increase intracellular calcium via activation of Gαq‐coupled G‐protein coupled receptors. Interestingly, expression of the Group 1 metabotropic glutamate receptors, mGluR1 and mGluR5, is not uniform across OPCs in the developing mouse hippocampus (Haberlandt et al., 2011) and NMDA receptor‐dependent calcium transients are only detected in ~20% of OPCs in the mouse optic nerve (Hamilton et al., 2010), suggesting that some alternative avenues for activity‐dependent calcium entry into OPCs may be restricted to subpopulations of OPCs.

## AUTHOR CONTRIBUTIONS

K.A.P., R.R., S.B., M.P., and R.G. performed the experiments and generated the figures. K.A.P., R.R., and KMY analyzed the data and wrote the manuscript. K.M.Y., R.G., L.F., and K.A.P. developed the project and obtained funding. J.C., K.A.P., and K.M.Y. provided supervision.

## Supporting information


**Figure S1** A Pdgfrα‐CreER BAC transgenic mouse line induces non‐specific recombination of the Tau‐mGFP transgene
*Pdgfrα‐CreER* transgenic mice (Kang et al., 2010) were crossed with cre‐sensitive *Tau‐mGFP* reporter mice. 30 μm brain cryosections from P60 + 7 and P60 + 30 *Pdgfrα‐CreER*::*Tau‐mGFP* double heterozygous offspring were immunostained to detect GFP (n = 10 mice total). a) Representative image of the forebrain of a P60 + 7 mouse to highlight the extensive non‐specific GFP‐labeling throughout the motor cortex and corpus callosum. A higher magnification image of the corpus callosum (b) and motor cortex (c). In these mice, it was not possible to identify individual mGFP‐labeled OPCs or newborn oligodendrocytes for quantification. This pattern of labeling was present in mice receiving Tx for 1, 2 or 4 consecutive days. This non‐specific recombination prevented the use of these mice for the lineage‐tracing of OPCs. Scale bars represent 150 μm (a) and 20 μm (b‐c).

**Figure S2** CaV1.2 deletion reduces K+ depolarisation‐induced calcium entry into adult OPCs
Acute brain slices were generated from adult (P60‐P90) control and CaV1.2‐deleted (*Pdgfrα‐CreER*:: *Cacna1c* ^*fl/fl*^) mice at Tx + 10 which were used to perform calcium imaging (with Fura‐2 a.m.) of GFP^+^ OPCs (a‐d) and GFP‐negative cells (e‐h) in the same slice. ΔF/F0 traces for GFP^+^ OPCs in control (i) and CaV1.2‐deleted (j) mice at baseline and following exposure to ACSF containing 50 mM K^+^ to induce depolarisation (gray traces are from individual cells and black trace shows the mean). (k) Quantification of ΔF/F0 between 8 and 10 mins for control OPCs in the absence or presence of nifedipine and CaV1.2‐deleted OPCs. (l‐m) ΔF/F0 traces for GFP‐negative cells in slices generated from control and CaV1.2‐deleted mice, before and after exposure to ACSF containing 50 mM K^+^ to induce depolarisation. (n) Quantification of ΔF/F0 between 8 and 10 mins for GFP‐negative cells in slices generated from control mice (+/− nifedipine) and CaV1.2‐deleted mice. o) Maximum ΔF/F0 for GFP^+^ OPCs and GFP‐negative cells in acute brain slices from control mice (+/− nifedipine treatment) and CaV1.2‐deleted mice. p) Quantification of the area under the curve (ΔF/F0*min) for GFP^+^ OPCs and GFP‐negative cells in acute brain slices generated from control mice (+/− nifedipine treatment) and CaV1.2‐deleted mice. Data is represented as the mean ± SEM for 5–20 cells per condition, from n = 3–6 mice per genotype. * p < 0.05, ** p < 0.01, *** p < 0.001 or **** *p* < 0.0001 for 1‐way (k, n) or 2‐way (o, p) ANOVA with Bonferroni's posttest. Scale bars represent 10 μm.

**Figure S3** CaV1.2 deletion enhances OPC proliferation in the superficial motor cortex
Control and CaV1.2‐deleted (*Pdgfrα‐CreER*:: *Cacna1c* ^*fl/fl*^) mice received EdU from P60 + 7 for 6 or 12 days before 30 μm brain cryosections were immunostained to detect EdU (green) and the OPC marker PDGFRα (red). (a‐b) Representative image of the motor cortex in control and CaV1.2‐deleted mice, indicating layers I, II/III, IV, V and VI. (c) Quantification of the proportion of OPCs in each layer of the motor cortex that have incorporated EdU after 6 days of labeling (P60 + 13). (d) Quantification of the proportion of OPCs in each layer of the motor cortex that have incorporated EdU after 12 days of labeling (P60 + 19). Data is represented as the mean ± SD of n = 5–7 mice per genotype per timepoint. * p < 0.05 and ** p < 0.01, 2‐way ANOVA with Bonferroni's posttest. Scale bars represent 100 μm.

**Figure S4** NG2‐labeled OPCs are lost from the corpus callosum of CaV1.2‐deleted mice
(a) Representative image of a P60 + 10 control (CTRL) mouse that received 2 consecutive i.p injections of EdU (green) to label proliferating PDGFRα^+^ OPCs (red) in the corpus callosum over a 4‐hour period. (b) Representative image of a P60 + 10 *Pdgfrα‐CreER*:: *Cacna1c* ^*fl/fl*^ (CaV1.2‐deleted) mouse that received 2 consecutive i.p injections of EdU (green) to label proliferating PDGFRα^+^ OPCs (red) in the corpus callosum over a 4‐hour period. (c) Quantification of the proportion of PDGFRα^+^ callosal control and CaV1.2‐deleted OPCs that became EdU‐labeled within 4‐hours at P60 + 10. Quantification was performed on a 30 μm confocal stack collected at the surface of a 200 μm vibratome slice. (d) Quantification of the number of PDGFRα^+^ OPC per mm^2^ in the corpus callosum of control and CaV1.2‐deleted mice at P60 + 10 (x‐y; fixed z‐depth of 30 μm from the surface of a 200 μm vibratome slice). (e‐f) Representative images of NG2 (green) and PDGFRα (red) labeling in the corpus callosum of P60 + 10 control and CaV1.2‐deleted mice. g) Quantification of the number of NG2^+^ cells and PDGFRα^+^ OPCs per mm^2^ (x‐y; fixed z‐depth of 30 μm cryosections) in the corpus callosum of control or CaV1.2‐deleted mice. Data is presented as the mean ± SD of n = 4–6 mice (c‐d) or n = 4 mice (g) for each genotype. Arrows indicate co‐labeled cells. * p < 0.05, unpaired t‐test. *** p < 0.001, 2‐way ANOVA with Bonferroni's posttest. Scale bars represent 10 μm (a‐b) and 20 μm (e‐f).

**Figure S5** Daily nimodipine delivery does not impact OPC density in the corpus callosum of adult mice.
Adult (P60) mice received daily injections of vehicle (5% ethanol/5% DMSO/40% polyethylene glycol 400/50% PBS) or 10 mg/kg nimodipine in vehicle (s.c.) for 4 or 7 consecutive days. Note that 4–7 days of pharmacological inhibition of L‐type VGCCs was selected to correspond to P60 + 7 to P60 + 10 in the CaV1.2 conditional deletion studies. 24 hours after the final injection mice were perfusion fixed and 30 μm cryosections prepared for immunostaining to detect the OPC marker PDGFRα (red) and the microglial marker IBA1 (green). Representative images show PDGFRα^+^ OPCs in the motor cortex of vehicle‐ (a‐b) or nimodipine‐treated (c‐d) mice. e) Quantification of PDGFRα^+^ OPCs density (per mm^2^; x‐y plane with fixed z‐depth of 30 μm) in the motor cortex of mice that received vehicle and nimodipine for 4 or 7 days. Representative images show PDGFRα^+^ OPCs in the corpus callosum of vehicle‐treated mice (f‐g) and nimodipine‐treated mice (h‐i). j) Quantification of PDGFRα^+^ OPCs density (per mm^2^; x‐y plane with fixed z‐depth of 30 μm) in the corpus callosum of mice that received vehicle or nimodipine for 4 or 7 days. Schampel et al. (2017) reported that nimodipine delivery resulted in the apoptotic loss of microglia from the spinal cord of mice with experimental autoimmune encephalomyelitis, but not from healthy mice. We confirm that nimodipine does not alter microglial number in the spinal cord or brain of healthy mice. k‐p) Representative images showing IBA1^+^ microglia in the motor cortex (k‐n), corpus callosum (l‐o) and spinal cord (m‐p) of mice that received vehicle or nimodipine for 7 days. q) Quantification of IBA1^+^ microglial density (per mm^2^; x‐y plane with fixed z‐depth of 30 μm) in the motor cortex, corpus callosum or spinal cord of mice that received vehicle or nimodipine for 7 days. Data is represented as the mean ± SD for n = 5 mice per treatment per timepoint. Scale bars represent 20 μm.

## Data Availability

The data that support the findings of this study are available from the corresponding author upon reasonable request.
