# Supplementary figures and images for: The voltage‐gated calcium channel CaV1.2 promotes adult oligodendrocyte progenitor cell survival in the mouse corpus callosum but not motor cortex
Source: Glia. 2019 Oct 12;68(2):376–92. doi: 10.1002/glia.23723 (PMC6916379; doi:10.1002/glia.23723)

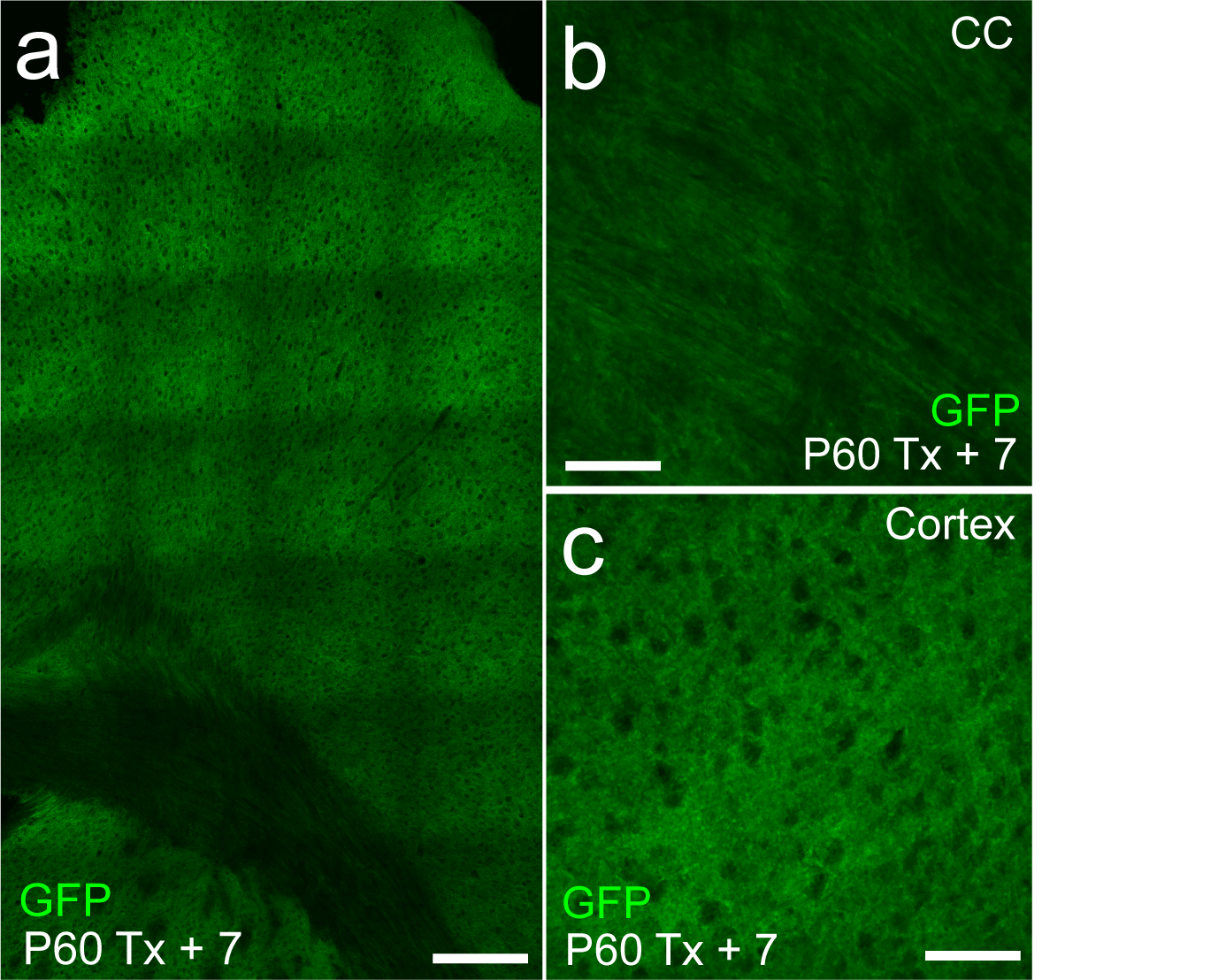

Supplement: Supplementary file 1 — Figure S1 A Pdgfrα‐CreER BAC transgenic mouse line induces non‐specific recombination of the Tau‐mGFP transgene Pdgfrα‐CreER transgenic mice (Kang et al., 2010) were crossed with cre‐sensitive Tau‐mGFP reporter mice. 30 μm brain cryosections from P60 + 7 and P60 + 30 Pdgfrα‐CreER::Tau‐mGFP double heterozygous offspring were immunostained to detect GFP (n = 10 mice total). a) Representative image of the forebrain of a P60 + 7 mouse to highlight the extensive non‐specific GFP‐labeling throughout the motor cortex and corpus callosum. A higher magnification image of the corpus callosum (b) and motor cortex (c). In these mice, it was not possible to identify individual mGFP‐labeled OPCs or newborn oligodendrocytes for quantification. This pattern of labeling was present in mice receiving Tx for 1, 2 or 4 consecutive days. This non‐specific recombination prevented the use of these mice for the lineage‐tracing of OPCs. Scale bars represent 150 μm (a) and 20 μm (b‐c). [file GLIA-68-376-s001.tif]

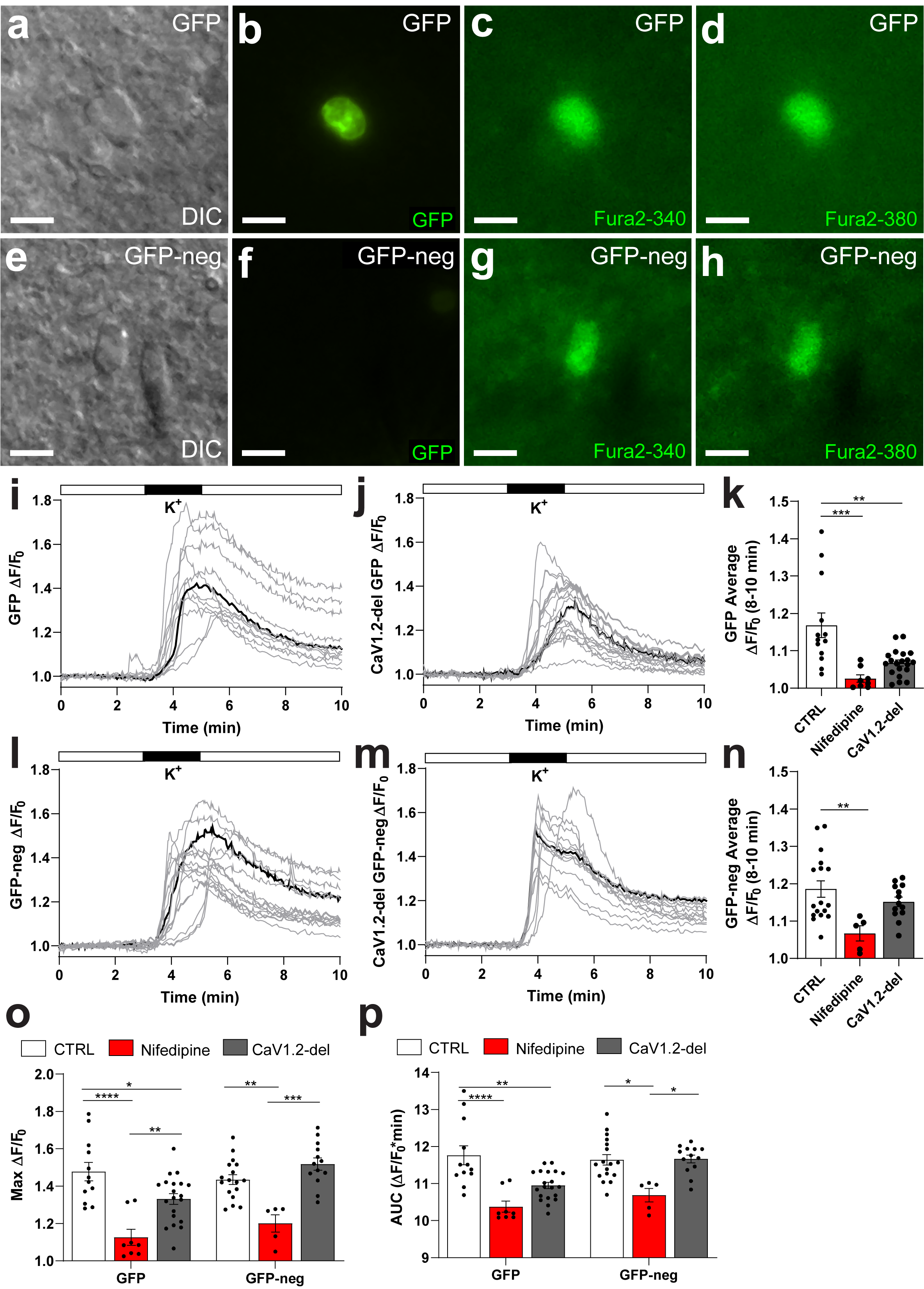

Supplement: Supplementary file 2 — Figure S2 CaV1.2 deletion reduces K+ depolarisation‐induced calcium entry into adult OPCs Acute brain slices were generated from adult (P60‐P90) control and CaV1.2‐deleted (Pdgfrα‐CreER:: Cacna1c fl/fl) mice at Tx + 10 which were used to perform calcium imaging (with Fura‐2 a.m.) of GFP+ OPCs (a‐d) and GFP‐negative cells (e‐h) in the same slice. ΔF/F0 traces for GFP+ OPCs in control (i) and CaV1.2‐deleted (j) mice at baseline and following exposure to ACSF containing 50 mM K+ to induce depolarisation (gray traces are from individual cells and black trace shows the mean). (k) Quantification of ΔF/F0 between 8 and 10 mins for control OPCs in the absence or presence of nifedipine and CaV1.2‐deleted OPCs. (l‐m) ΔF/F0 traces for GFP‐negative cells in slices generated from control and CaV1.2‐deleted mice, before and after exposure to ACSF containing 50 mM K+ to induce depolarisation. (n) Quantification of ΔF/F0 between 8 and 10 mins for GFP‐negative cells in slices generated from control mice (+/− nifedipine) and CaV1.2‐deleted mice. o) Maximum ΔF/F0 for GFP+ OPCs and GFP‐negative cells in acute brain slices from control mice (+/− nifedipine treatment) and CaV1.2‐deleted mice. p) Quantification of the area under the curve (ΔF/F0*min) for GFP+ OPCs and GFP‐negative cells in acute brain slices generated from control mice (+/− nifedipine treatment) and CaV1.2‐deleted mice. Data is represented as the mean ± SEM for 5–20 cells per condition, from n = 3–6 mice per genotype. * p < 0.05, ** p < 0.01, *** p < 0.001 or **** p < 0.0001 for 1‐way (k, n) or 2‐way (o, p) ANOVA with Bonferroni's posttest. Scale bars represent 10 μm. [file GLIA-68-376-s002.tif]

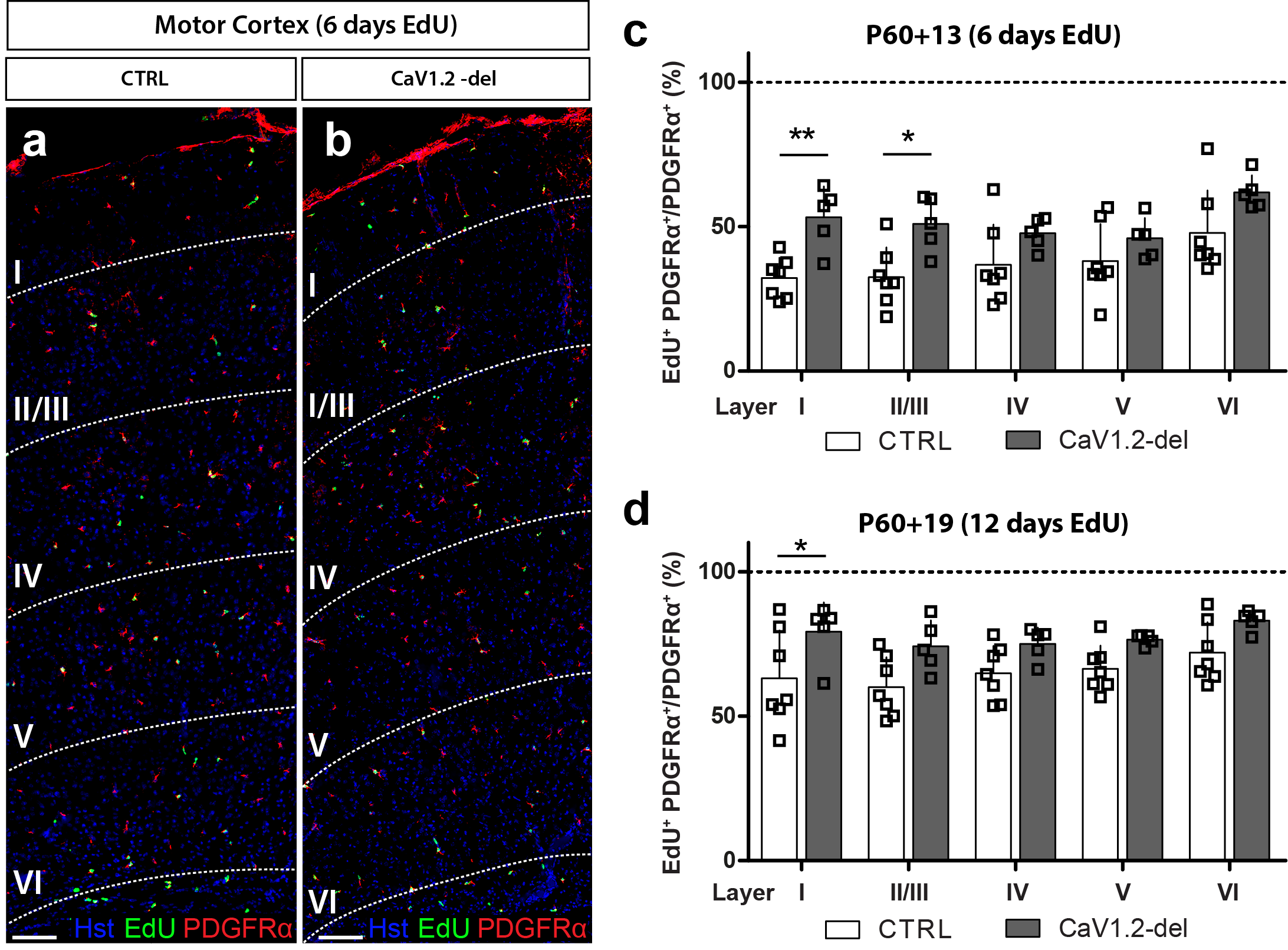

Supplement: Supplementary file 3 — Figure S3 CaV1.2 deletion enhances OPC proliferation in the superficial motor cortex Control and CaV1.2‐deleted (Pdgfrα‐CreER:: Cacna1c fl/fl) mice received EdU from P60 + 7 for 6 or 12 days before 30 μm brain cryosections were immunostained to detect EdU (green) and the OPC marker PDGFRα (red). (a‐b) Representative image of the motor cortex in control and CaV1.2‐deleted mice, indicating layers I, II/III, IV, V and VI. (c) Quantification of the proportion of OPCs in each layer of the motor cortex that have incorporated EdU after 6 days of labeling (P60 + 13). (d) Quantification of the proportion of OPCs in each layer of the motor cortex that have incorporated EdU after 12 days of labeling (P60 + 19). Data is represented as the mean ± SD of n = 5–7 mice per genotype per timepoint. * p < 0.05 and ** p < 0.01, 2‐way ANOVA with Bonferroni's posttest. Scale bars represent 100 μm. [file GLIA-68-376-s003.tif]

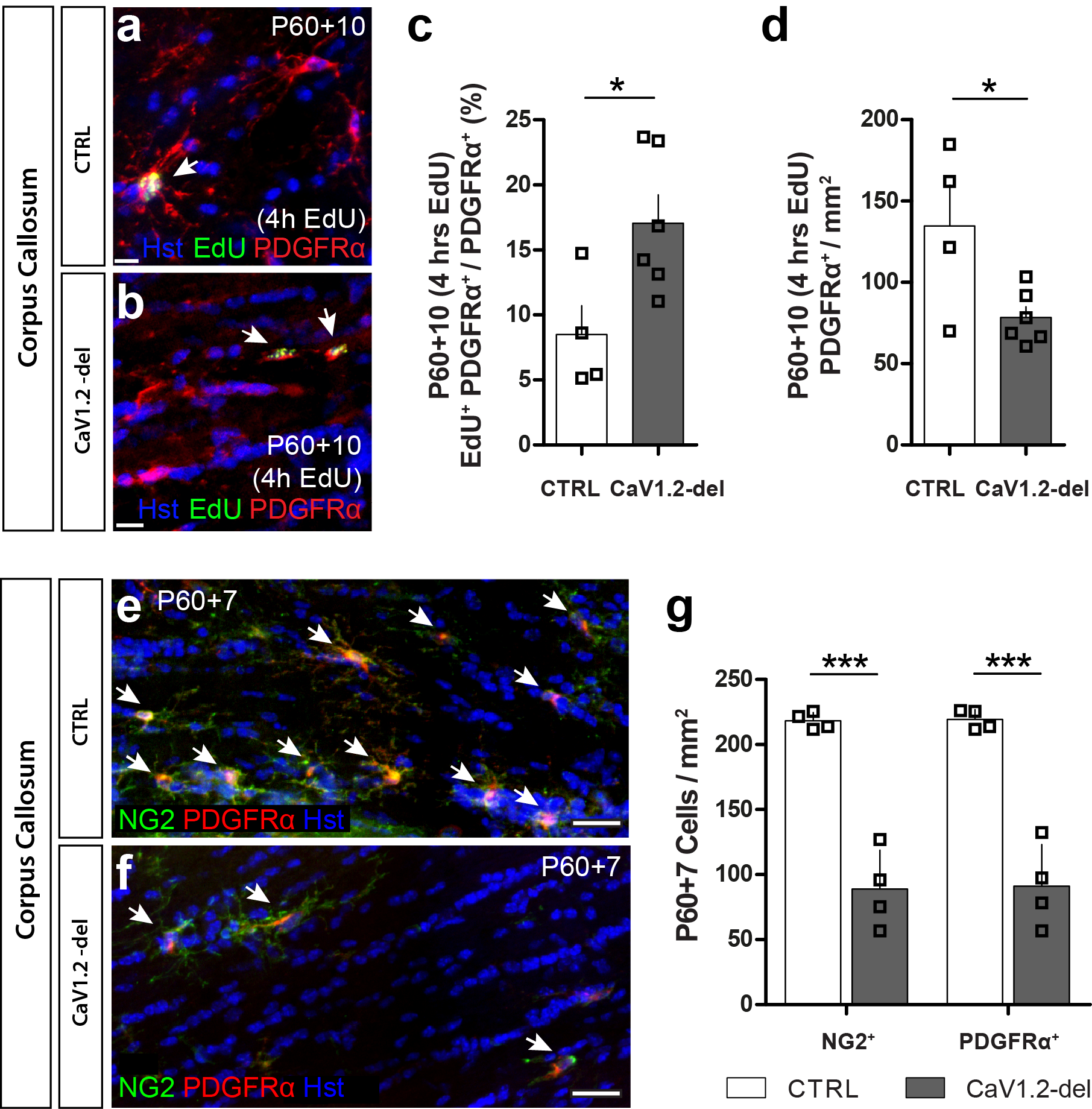

Supplement: Supplementary file 4 — Figure S4 NG2‐labeled OPCs are lost from the corpus callosum of CaV1.2‐deleted mice (a) Representative image of a P60 + 10 control (CTRL) mouse that received 2 consecutive i.p injections of EdU (green) to label proliferating PDGFRα+ OPCs (red) in the corpus callosum over a 4‐hour period. (b) Representative image of a P60 + 10 Pdgfrα‐CreER:: Cacna1c fl/fl (CaV1.2‐deleted) mouse that received 2 consecutive i.p injections of EdU (green) to label proliferating PDGFRα+ OPCs (red) in the corpus callosum over a 4‐hour period. (c) Quantification of the proportion of PDGFRα+ callosal control and CaV1.2‐deleted OPCs that became EdU‐labeled within 4‐hours at P60 + 10. Quantification was performed on a 30 μm confocal stack collected at the surface of a 200 μm vibratome slice. (d) Quantification of the number of PDGFRα+ OPC per mm2 in the corpus callosum of control and CaV1.2‐deleted mice at P60 + 10 (x‐y; fixed z‐depth of 30 μm from the surface of a 200 μm vibratome slice). (e‐f) Representative images of NG2 (green) and PDGFRα (red) labeling in the corpus callosum of P60 + 10 control and CaV1.2‐deleted mice. g) Quantification of the number of NG2+ cells and PDGFRα+ OPCs per mm2 (x‐y; fixed z‐depth of 30 μm cryosections) in the corpus callosum of control or CaV1.2‐deleted mice. Data is presented as the mean ± SD of n = 4–6 mice (c‐d) or n = 4 mice (g) for each genotype. Arrows indicate co‐labeled cells. * p < 0.05, unpaired t‐test. *** p < 0.001, 2‐way ANOVA with Bonferroni's posttest. Scale bars represent 10 μm (a‐b) and 20 μm (e‐f). [file GLIA-68-376-s004.tif]

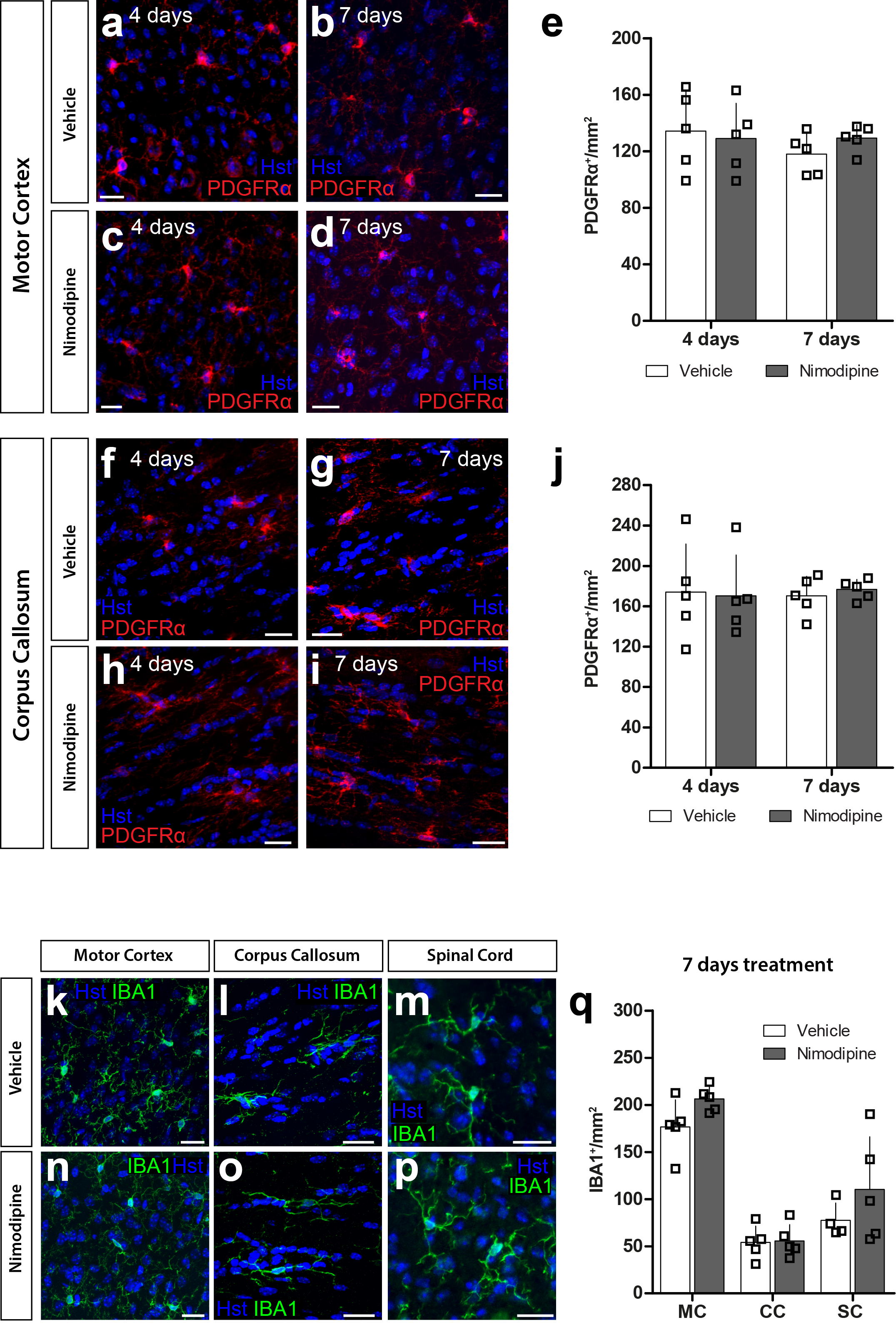

Supplement: Supplementary file 5 — Figure S5 Daily nimodipine delivery does not impact OPC density in the corpus callosum of adult mice. Adult (P60) mice received daily injections of vehicle (5% ethanol/5% DMSO/40% polyethylene glycol 400/50% PBS) or 10 mg/kg nimodipine in vehicle (s.c.) for 4 or 7 consecutive days. Note that 4–7 days of pharmacological inhibition of L‐type VGCCs was selected to correspond to P60 + 7 to P60 + 10 in the CaV1.2 conditional deletion studies. 24 hours after the final injection mice were perfusion fixed and 30 μm cryosections prepared for immunostaining to detect the OPC marker PDGFRα (red) and the microglial marker IBA1 (green). Representative images show PDGFRα+ OPCs in the motor cortex of vehicle‐ (a‐b) or nimodipine‐treated (c‐d) mice. e) Quantification of PDGFRα+ OPCs density (per mm2; x‐y plane with fixed z‐depth of 30 μm) in the motor cortex of mice that received vehicle and nimodipine for 4 or 7 days. Representative images show PDGFRα+ OPCs in the corpus callosum of vehicle‐treated mice (f‐g) and nimodipine‐treated mice (h‐i). j) Quantification of PDGFRα+ OPCs density (per mm2; x‐y plane with fixed z‐depth of 30 μm) in the corpus callosum of mice that received vehicle or nimodipine for 4 or 7 days. Schampel et al. (2017) reported that nimodipine delivery resulted in the apoptotic loss of microglia from the spinal cord of mice with experimental autoimmune encephalomyelitis, but not from healthy mice. We confirm that nimodipine does not alter microglial number in the spinal cord or brain of healthy mice. k‐p) Representative images showing IBA1+ microglia in the motor cortex (k‐n), corpus callosum (l‐o) and spinal cord (m‐p) of mice that received vehicle or nimodipine for 7 days. q) Quantification of IBA1+ microglial density (per mm2; x‐y plane with fixed z‐depth of 30 μm) in the motor cortex, corpus callosum or spinal cord of mice that received vehicle or nimodipine for 7 days. Data is represented as the mean ± SD for n = 5 mice per treatment per timepoint. Scale ba [file GLIA-68-376-s005.tif]
